# Puf Mediates Translation Repression of Transmission-Blocking Vaccine Candidates in Malaria Parasites

**DOI:** 10.1371/journal.ppat.1003268

**Published:** 2013-04-18

**Authors:** Jun Miao, Qi Fan, Daniel Parker, Xiaolian Li, Jianyong Li, Liwang Cui

**Affiliations:** 1 Department of Entomology, Pennsylvania State University, University Park, Pennsylvania, United States of America; 2 Dalian Institute of Biotechnology, Dalian, Liaoning Province, China; 3 Department of Biochemistry, Virginia Tech, Blacksburg, Virginia, United States of America; Zentrum für Molekulare Biologie der Universität Heidelberg (ZMBH), Germany

## Abstract

Translational control of gene expression plays an essential role in development. In malaria parasites, translational regulation is critical during the development of specialized transition stages between the vertebrate host and mosquito vector. Here we show that a Pumilio/FBF (Puf) family RNA-binding protein, PfPuf2, is required for the translation repression of a number of transcripts in gametocytes including two genes encoding the transmission-blocking vaccine candidates Pfs25 and Pfs28. Whereas studies to date support a paradigm of Puf-mediated translation regulation through 3′ untranslated regions (UTRs) of target mRNAs, this study, for the first time, identifies a functional Puf-binding element (PBE) in the 5′UTR of *pfs25*. We provide both *in vitro* and *in vivo* evidence to demonstrate that PfPuf2 binds to the PBEs in *pfs25* and *pfs28* to mediate translation repression. This finding provides a renewed view of Pufs as versatile translation regulators and sheds light on their functions in the development of lower branches of eukaryotes.

## Author Summary

*Plasmodium falciparum* is the causative agent of the deadliest malaria, which causes over one million deaths each year. The malaria parasite completes its life cycle in two hosts, mosquitoes and humans. Gametocytes, the sexual form of the malaria parasite, mediate transmission from humans to mosquitoes. Many transcripts, including the two transmission-blocking vaccine (TBV) candidates Pfs25 and Pfs28, are translationally regulated during gametocyte formation and transmission, but the molecular mechanisms of translation regulation are unknown. Our study revealed that Puf2, an RNA-binding protein, controls the translation of a number of transcripts in gametocytes, including the two TBV candidates, by binding target mRNAs at Puf-binding elements (PBE) in the 3′ untranslated regions (UTR) of *Pfs28* and 5′ UTR of *Pfs25*. Our study provides solid evidence demonstrating that a Puf family member serves as a translation regulator of a number of transcripts during the transition stage between a human host and a mosquito vector, and for the first time, identifies a functional PBE in the 5′ UTR of a transcript.

## Introduction

Translational control plays a pivotal role in the regulation of gene expression in most organisms. It allows the cell to respond more rapidly to external stimuli than transcriptional regulation [1]. Translational control is especially critical during early development in metazoans, since early embryonic development occurring in the absence of transcription relies on the stockpiled maternal mRNAs, whose translation must be blocked during their synthesis and deposition, but later activated in response to fertilization [2], [3]. Translational control is achieved through a network of RNA binding proteins (RBPs) that regulate mRNA stability, localization and translatability [4]. Extensive studies in metazoans have identified a unifying theme of translational regulation, which involves the interactions of RBPs with *cis*-acting sequence elements located most often in 3′ untranslated regions (UTRs) of the mRNAs [5]–[7]. A prominent example is the Puf (**Pu**milio and **f**em-3 binding factor) family RBPs, which are ubiquitously present throughout all eukaryotic branches [5], [8]. Puf proteins are defined by a conserved RNA-binding domain, referred to as the Pumilio homology domain, which contains eight α-helical repeats of ∼38 amino acids and folds in an arc shape [9], [10]. Pufs use the concave surface to bind the Puf-binding elements (PBEs) in target mRNAs, which typically leads to translation repression, deadenylation, and/or mRNA decay [11]–[13], or in some cases, translation activation [14], [15]. The canonical PBE comprises a core sequence ‘UGUX_3–5_UA’ located in the 3′UTRs of most Puf target mRNAs characterized thus far, lending strong support of a paradigm of Puf regulation through the 3′UTRs [16]–[20].

Despite that Puf-RNA interactions use a conserved molecular mechanism [21], Puf proteins have a large repertoire of mRNA targets with diverse cellular functions [8]. Studies in model organisms have shown that Pufs participate in an array of cellular processes including differentiation and development, germline stem cell homeostasis, neuronal function, mitochondrial biogenesis, and rRNA maturation and ribosome assembly [5], [8], [22]–[24]. Based on a converging theme of functions of Pufs in development, it has been speculated that the ancestral role of Pufs is to maintain proliferation and repress differentiation [5]. To date, our understanding of the functions of the Puf family is mostly obtained from studies in metazoans and model organisms, while their functions in lower domains of unicellular eukaryotes are relatively less explored.

The life cycle of the malaria parasites *Plasmodium* alternates between mosquito vectors and vertebrate hosts. Transitions between the hosts are carried out by highly specialized stages. Transmission from the vertebrate host to the mosquito requires the sexual forms, or gametocytes, which are formed in erythrocytes of the vertebrate host. In the mosquito, parasites undergo sporogonic development to produce sporozoites, which are injected into the vertebrate host during mosquito feeding to initiate infection of the liver. These host transition events depend on translational activation of stored, silent mRNAs in gametocytes and sporozoites, respectively. For example, the mRNAs encoding the major ookinete surface proteins Pbs25and Pbs28 in the rodent malaria *Plasmodium berghei* are deposited in gametocytes in a translationally repressed (TR) form [25], [26]. Similarly, although the mRNAs of *pfs25* and *pfs28* are already present in *P. falciparum* gametocytes, synthesis of these proteins occurs predominantly in ookinetes [27]–[29], suggesting post-transcriptional regulation. In particular, mass spectrometry surveys of *P. falciparum* gametocyte proteomes have detected Pfs25 protein in gametocytes [30], [31]. Comparison of the gametocyte transcriptome and proteome in *P. berghei* has identified an additional nine TR transcripts, of which eight have orthologs in *P. falciparum* [32]. The stability of these “maternal” mRNAs in the gametocytes appears to be conferred by a protein complex containing the DEAD-box RNA helicase DOZI [33]; deletion of either DOZI or CITH of this complex led to substantial losses of the maternal transcripts including the *pbs25* and *pbs28* mRNAs [33], [34]. Further analysis reveals that translational repression of the TR transcripts in *P. berghei* gametocytes is mediated by 47-nucleotide U-rich elements that are localized in the 5′ and 3′ UTRs of these mRNAs [32], [35], but the *trans* element(s) that mediates translation repression through the U-rich elements remains elusive.

Two conserved Puf proteins in *Plasmodium* are preferentially expressed in gametocyte and sporozoite stages [36], [37]. Functional studies of Puf1 did not reveal noticeable phenotypic changes in *P. berghei* [38], [39]. Puf2, however, appears to play important roles in both gametocyte and sporozoite stages, and the functions of Puf2 seem to conform to the presumptive ancestor role of the Puf family in promoting proliferation and suppressing differentiation. Genetic knockout of the Puf2 gene (ΔPuf2) in *P. falciparum* and *P. berghei* promotes differentiation of gametocytes and elevates the male/female sex ratio [38], [40]. However, this finding from Δ*Puf2* is not universal in *P. berghei*, as this phenotypic change was examined but not found in another study [39]. In *P. berghei* sporozoites, ΔPuf2 results in earlier expression of a set of genes that are otherwise expressed in the subsequent hepatic stages, which leads to premature transformation of the sporozoites into forms resembling early intra-hepatic stages inside the salivary glands of the mosquito [38], [39]. Among these down-regulated genes is the protein kinase IK2, which phosphorylates the translation initiation factor eIF2α and down-regulates protein translation in sporozoites. This partially explains why the phenotype of ΔPuf2 mirrors that of the *IK2* gene deletion [41]. These findings strongly suggest that Puf2 is a key translational regulator during the two host transition stages, but the molecular mechanism whereby Puf2 regulates this process is unclear.

To elucidate the molecular mechanisms of translational regulation during the developmental transition in the human malaria parasite *P. falciparum*, we characterized the functions of Puf proteins during the formation of gametocytes, or gametocytogenesis. By comparing the gametocyte transcriptomes between the wild-type and ΔPuf2 using microarray analysis, we identify a number of putative TR transcripts, including the two encoding transmission-blocking vaccine (TBV) candidates Pfs25 and Pfs28, which are upregulated in ΔPuf2 gametocytes. We show that PfPuf2 is the translational repressor of *pfs25* and *pfs28* and translational repression is mediated through the binding of PfPuf2 to PBEs in the UTRs of these genes. However, while all PBEs characterized to date are localized in the 3′UTR or 3′UTR proximal coding region of target mRNAs, we identify the location of a functional PBE in the 5′UTR. This finding changes our view of the paradigm of Puf regulation solely through PBEs localized in the 3′UTRs, suggesting that Puf proteins are versatile translation regulators and act through PBEs located in both 3′ and 5′UTRs.

## Results

### Deletion of *PfPuf2* results in dramatic changes in gametocyte transcriptomes

**Figure 1:**
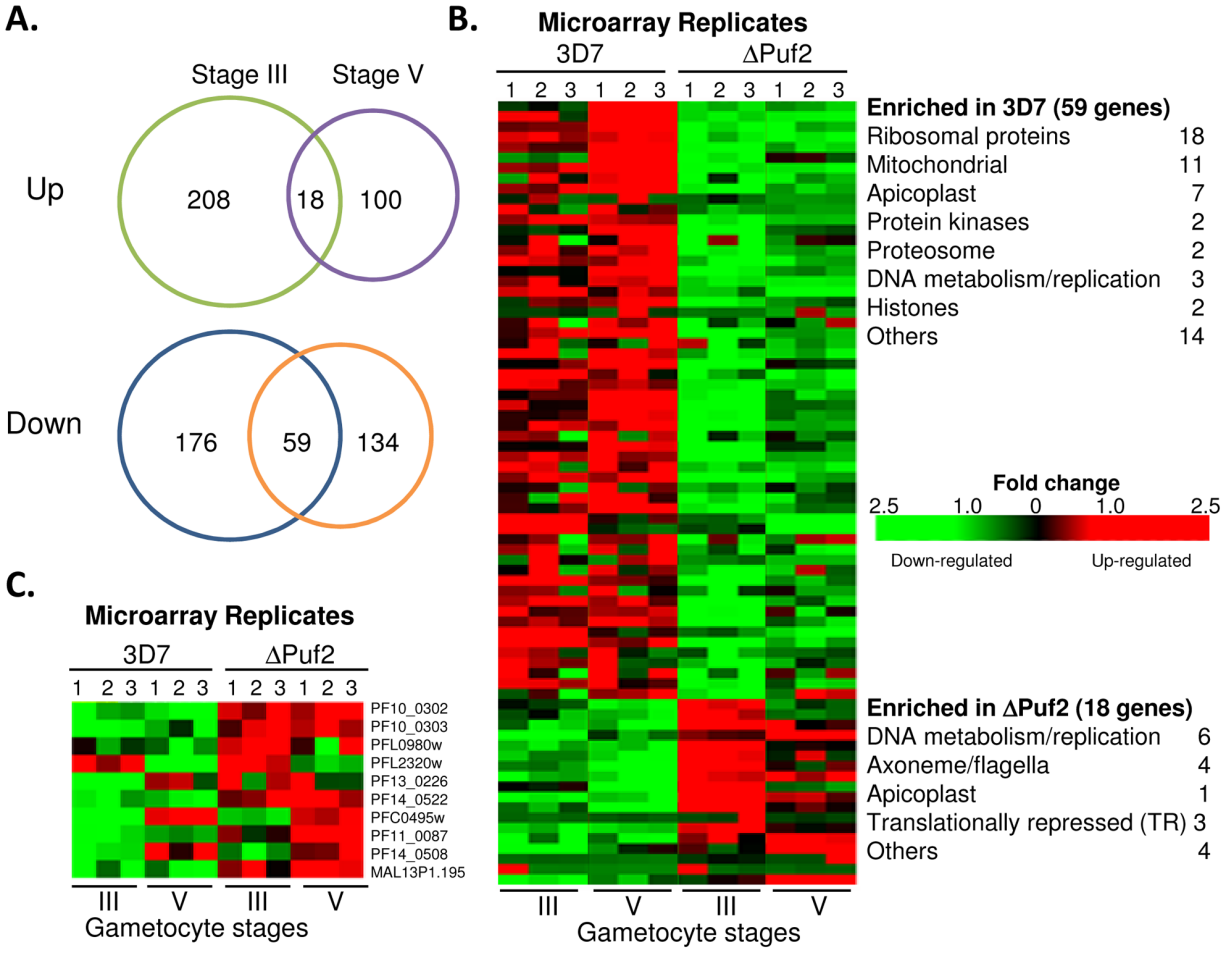
PfPuf2 deletion and genome-wide changes in gametocyte transcriptomes. (**A**) Venn diagrams of up- or down-regulated genes in stage III and V gametocytes in ΔPuf2 line as compared to those in wild-type 3D7 line. (**B**) Heat map of consistently up- and down-regulated transcripts in stage III and V gametocytes in 3D7 and ΔPuf2. For each parasite line and at each time points, microarray experiments were performed in triplicates (numbers 1–3). Functional categories of genes enriched in 3D7 (59) and ΔPuf2 (18) are listed. The scale bar shows fold changes in mRNA levels. (**C**) Heat map showing the mRNA levels in 3D7 and ΔPuf2 of *P. falciparum* orthologs of TR transcripts identified in *P. berghei* gametocytes.

Puf proteins, as translational repressors, affect the stability and abundance of target mRNAs. To identify potential target genes of PfPuf2 in *P. falciparum* gametocytes, we compared the transcriptomes in stage III (8-day-old) and stage V (12-day-old) gametocytes between wild-type 3D7 and the ΔPuf2 line by microarray analysis. A multi-stage process was used for identifying genes that showed significant changes in transcript levels between 3D7 and ΔPuf2. Analysis based on *t*-statistics from the microarray data identified 226 and 118 up-regulated transcripts as well as 235 and 193 down-regulated transcripts in ΔPuf2 in stage III and V gametocytes, respectively (Figure 1A, Table S1A and B). In ΔPuf2, the two gametocyte stages shared 18 up-regulated and 59 down-regulated genes, respectively (Figure 1A, Table S1C). This result was further verified using fold changes of average transcription levels and the Significance Analysis of Microarray (SAM) method [42], which showed >60% agreement rate in the most significant results with the *t*-statistics (Table S1). Collectively, deletion of *PfPuf2* had a considerable effect on global transcript levels with ∼13% of genes in the genome showing substantial perturbation in mRNA levels in stage III and V gametocytes. Functional classification of the genes with significant changes in mRNA levels revealed a significant enrichment of genes associated with different sexes of the *Plasmodium* gametocytes [43], consistent with the elevated male/female sex ratio in ΔPuf2 [40]. Specifically, there was a significant enrichment of genes associated with the functions of ribosomes and mitochondria in 3D7, whereas genes involved in DNA replication/metabolism and axoneme formation were enriched in ΔPuf2 gametocytes (Figure 1B, Table S1). Remarkably, of the 18 genes enriched in ΔPuf2 gametocytes three belong to the group of TR transcripts found in *Plasmodium* gametocytes, including the TBV candidates *pfs25* and *pfs28* [32]. Pfs25 and Pfs28 are both female-specific, and their mRNAs are deposited in gametocytes but prominent protein translation occurs in gametes, zygotes and ookinetes [27]–[29]. This result prompted us to analyze the *P. falciparum* orthologs of the eight additional TR transcripts identified in *P. berghei* gametocytes [32]. We found that almost all putative TR transcripts were up-regulated in ΔPuf2, despite that many of these TR transcripts are female-specific and that the ΔPuf2 line is more male-biased (Figure 1C). The increased abundance of putative TR transcripts in the ΔPuf2 line is in agreement with the functions of Puf family proteins in repressing translation and promoting mRNA decay [11]–[13].

In *P. berghei* gametocytes, DOZI and CITH are involved in the protection of translationally quiescent maternal mRNAs [33], [34]. Deletion of either *pbdozi* or *pbcith* resulted in a >2-fold down-regulation of a common set of 117 mRNAs in gametocytes. Comparison of the up-regulated transcripts in ΔPuf2 stage III and V gametocytes with these 117 transcripts identified only 11 and 8 common transcripts, respectively (Table S1D), which include 7 of the 10 TR transcripts identified earlier in *P. berghei* gametocytes.

### Deletion of *PfPuf2* leads to de-repression of TR transcripts

**Figure 2:**
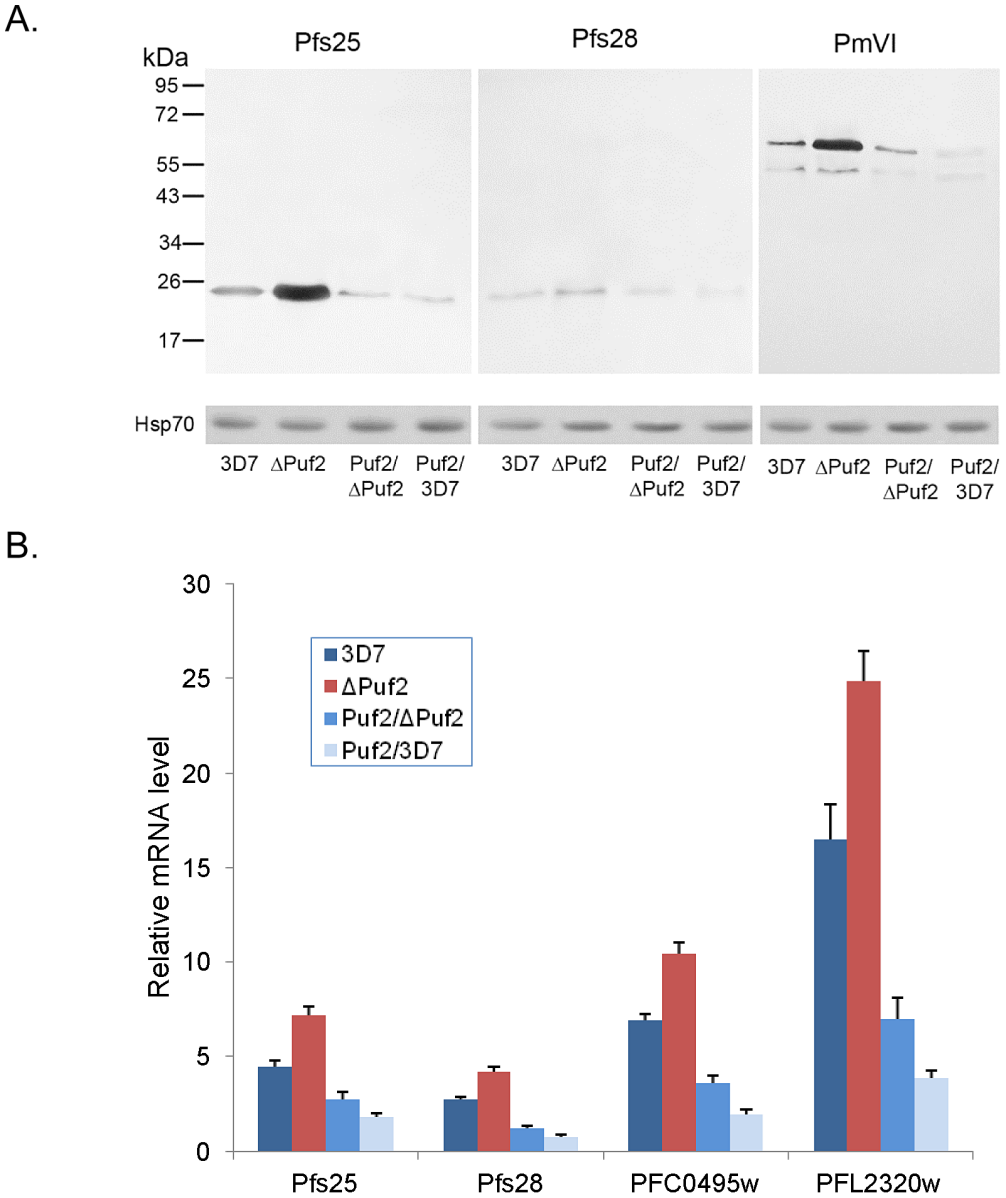
The effect of PfPuf2 levels on the expression of Pfs25, Pfs28 and PmVI. (**A**) Western blot comparison of protein abundance of Pfs25, Pfs28 and PmVI in mature gametocytes in 3D7, ΔPuf2, ΔPuf2 with PfPuf2 overexpression, and 3D7 with PfPuf2 overexpression. PfPuf2 overexpression was driven by the constitutive *hsp86* promoter from an episomal plasmid. Western blot of Hsp70 protein was used as loading controls. (**B**) Real-time RT-PCR analysis of transcript abundance of *pfs25*, *pfs28*, *PmVI* (*PFC0495w*) and *PFL2320w* in 3D7, ΔPuf2, ΔPuf2 with PfPuf2 overexpression, and 3D7 with PfPuf2 overexpression. The relative mRNA levels of these genes were normalized using *PF07_0073* as an internal reference.

To determine whether the elevated mRNA levels of the putative TR transcripts in ΔPuf2 gametocytes are correlated with increased protein expression, we compared the protein and mRNA levels of Pfs25 (PF10_0303), Pfs28 (PF10_0302), and plasmepsin VI (PmVI, PFC0495w) in gametocytes between 3D7, ΔPuf2, and two transgenic parasite lines with episomal overexpression of PfPuf2. Western blots of protein extracts from stage V gametocytes using antibodies against these three proteins detected higher levels of protein expression in ΔPuf2 than in 3D7 (Figure 2A). Moreover, episomal overexpression of PfPuf2 in both ΔPuf2 and 3D7 backgrounds further drove down the protein levels of Pfs25, Pfs28 and PmVI, suggesting a function of PfPuf2 in translational repression of these transcripts. Real-time RT-PCR analysis of four TR transcripts (*pfs28*, *pfs25*, *PmVI* and *PFL2320w*) also detected higher mRNA levels in ΔPuf2 than wild-type 3D7, whereas PfPuf2 overexpression further reduced the transcript levels of these genes (Figure 2B). This result suggests that PfPuf2 might be associated with increased degradation of these TR transcripts and that the action of PfPuf2 might be dosage-dependent.

### PfPuf2 is associated with TR transcripts

**Figure 3:**
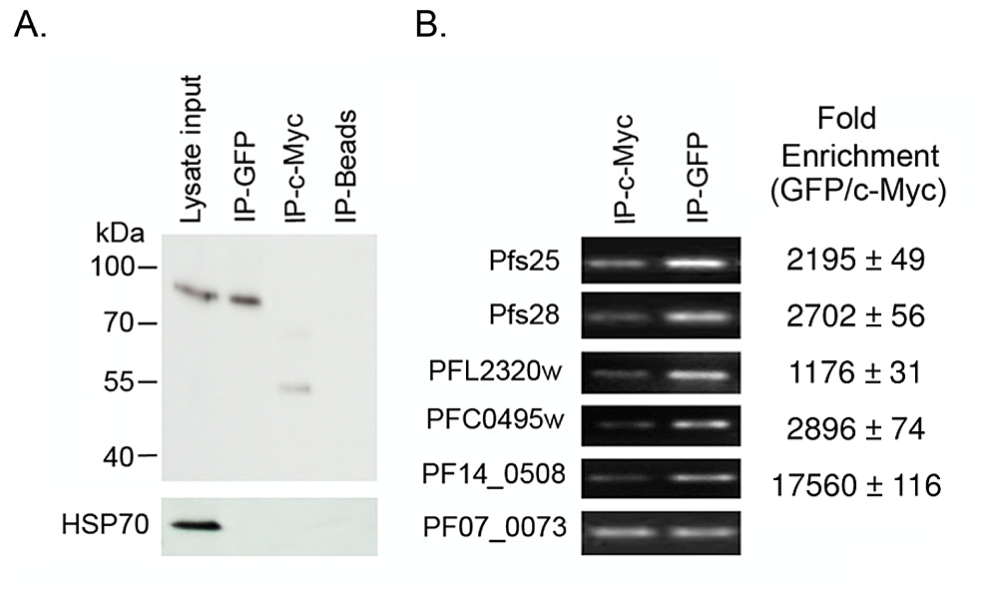
Association of PfPuf2 with putative TR mRNAs in gametocytes. (**A**) Western blot analysis of immunoprecipitations (IPs) of mature gametocyte lysates from the PfPuf2-GFP line with the anti-GFP antibodies (upper panel) or anti-HSP70 antibody (lower panel). Shown here are lysate input, IP with the anti-GFP antibodies (IP-GFP), IP with the anti-c-Myc antibodies (IP-c-Myc), and the beads only (IP-Beads). (**B**) RT-PCR analysis of RNA extracted from the IP-GFP and IP-c-Myc shown in (A). Included in the RT-PCR comparison were *pfs25* and *pfs28* and three other putative TR transcripts, with *PF07_0073* serving as an internal control. Left panel shows the semi-quantitative RT-PCR analysis with 35 PCR cycles, while right panel shows fold enrichment of corresponding transcripts ( = relative mRNA level from IP-GFP/relative mRNA level from IP-c-Myc). The RT-PCR results were calculated from three experiments and the values of fold enrichment are the means ± standard deviations.

To directly demonstrate that PfPuf2 was associated with the mRNAs of the TR transcripts, immunoprecipitations (IPs) were performed using a monoclonal antibody against either green fluorescent protein (GFP) or c-Myc (as a control) with lysates of mature gametocytes from the PfPuf2-GFP parasite line [40]. Western blot showed that PfPuf2-GFP was only present in the eluate with the GFP antibody, whereas the control protein Hsp70 was found only in the input lysate (Figure 3A). We selected several putative TR transcripts for quantitation to see whether they were associated with PfPuf2. RT-PCR analysis showed noticeably higher levels of transcripts of *pfs25*, *pfs28*, *PFL2320w*, *PFC0495w* and *PF14_0508* in the GFP eluate than in the c-Myc eluate (Figure 3B), and quantitation by real-time RT-PCR showed >1100-fold enrichment of mRNAs of these putative TR transcripts in the GFP eluate versus the c-Myc control eluate (Figure 3B). This result suggests that PfPuf2 and transcripts of the analyzed TR transcripts were present in the same mRNA-protein complex(es).

### Flanking regions (FRs) of TR transcripts contain *cis*-regulatory elements of translation repression

**Figure 4:**
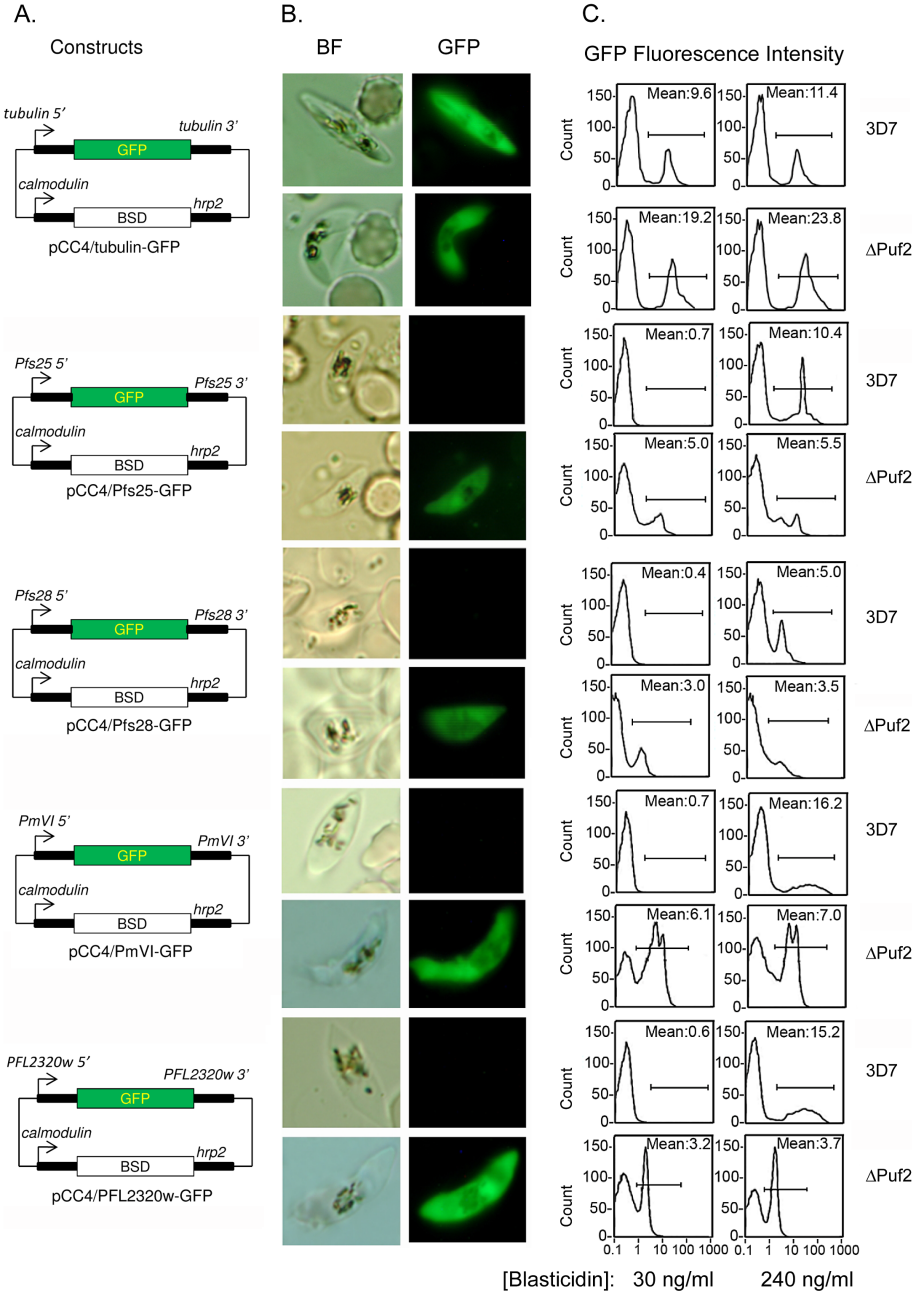
Loss of translational repression of the GFP reporter in the ΔPuf2 line. (**A**) Schematics of constructs for transfection of the 3D7 and ΔPuf2 lines based on the pCC4 vector. The *gfp* ORF was flanked by 5′ and 3′ FRs of *α-tubilin II* (control), *pfs25*, *pfs28*, *pmVI* and *pfl2320w*. (**B**) Representative bright-field (BF) and fluorescent (GFP) microscopic images of gametocytes in transfected 3D7 and ΔPuf2 under selection of 30 ng/ml blasticidin. (**C**) Quantitation of GFP-positive gametocytes by flow cytometry. Transgenic parasites were cultured under two drug concentrations of 30 and 240 ng/ml of blasticidin, respectively. The fluorescence intensity of gametocytes was measured and the mean fluorescence intensity was calculated from ∼2000 purified gametocytes.

To examine whether the UTRs of the TR transcripts mediate translational repression and to further implicate PfPuf2 as the translation repressor, we analyzed the four putative TR transcripts (*pfs28*, *pfs25*, *PmVI* and *PFL2320w*) and the male-specific *α-tubulin II* gene as a non-repressed control using a GFP reporter system. By rapid amplification of cDNA ends (RACE), we determined that all four TR transcripts had relatively short 5′UTRs (143–306 nucleotides) and 3′UTRs (202–401 nucleotides) (Figure S1). To ensure that functional promoters and polyadenylation sites are included in the reporter plasmids, ∼1 kb fragments from the 5′ and 3′ FRs of the four TR genes and *α-tubulin II* were cloned to flank the GFP open reading frame (ORF) (Figure 4A). Both 3D7 and ΔPuf2 lines were transfected with the reporter plasmids and selected with 30 ng/ml of blasticidin. GFP expression in gametocytes was monitored by fluorescent microscopy and quantified by flow cytometry. Consistent with an earlier report, the 1 kb 5′ FR of *α-tubullin II* is able to direct gametocyte-specific expression [44]. Gametocyte-specific expression was also observed for the *pfs28*, *pfs25*, *PmVI* and *PFL2320w* constructs (data not shown). In 3D7 parasite transfected with the control reporter pCC4/tubulin-GFP, GFP fluorescence was easily seen under a fluorescent microscope and GFP signal intensity of purified gametocytes was relatively high (Figure 4B,C), suggesting that the GFP transcript with the *α-tubulin II* UTRs was not translationally blocked in the wild-type parasite. Transfection of the ΔPuf2 parasites resulted in an increase of the fluorescence intensity of purified gametocytes, which is consistent with increased male∶female ratio in ΔPuf2 [40]. In contrast, GFP-positive gametocytes were rarely observed in 3D7 transfected with the four TR constructs (<1%), but much more abundant in transfected ΔPuf2 (>25%) (Figure 4B). GFP signal of purified gametocytes was also significantly stronger in transfected ΔPuf2 parasites (*P* = 0.00018, *t*-test) (Figure 4C). This result indicates that the FRs of all four putative TR transcripts contain *cis* elements for translation repression of the GFP reporter in 3D7; however, this translation repression was relieved in ΔPuf2. To verify that the lower level of GFP expression in transfected 3D7 parasites was due to translational repression of the *gfp* mRNAs by PfPuf2, we attempted to alter the molar ratios of *gfp* mRNA/PfPuf2 protein by culturing the transfected parasites at 240 ng/ml of blasticidin. Increased drug pressure increases the copy numbers of the episomal plasmids, which in turn, results in higher transcription of the reporter RNA [40], [45]. Indeed, the *gfp* mRNA levels in transfected 3D7 parasites were substantially increased under the higher drug selection pressure (Figure S2). In both 3D7 and ΔPuf2 parasites transfected with the non-repressed control *α-tubulin II* construct, the increase of drug selection pressure only slightly elevated the mean GFP signal intensity of purified gametocytes. In stark contrast, in 3D7 lines transfected with the TR constructs, a significant increase in the GFP fluorescence intensity of purified gametocytes was detected under the higher drug concentration (*P* = 0.00066, *t*-test) (Figure 4C). However, the increase of drug selection pressure had almost no effect on GFP signal intensity in gametocytes in transfected ΔPuf2 (*P* = 0.613, *t*-test). These results demonstrated a loss of translation repression of the reporter constructs in the ΔPuf2 line and further implicated a dosage-dependent effect of PfPuf2 on translation repression of these TR transcripts in *P. falciparum* gametocytes.

### *Cis*-regulatory elements are located in the 5′ and 3′ UTRs of the TR transcripts

**Figure 5:**
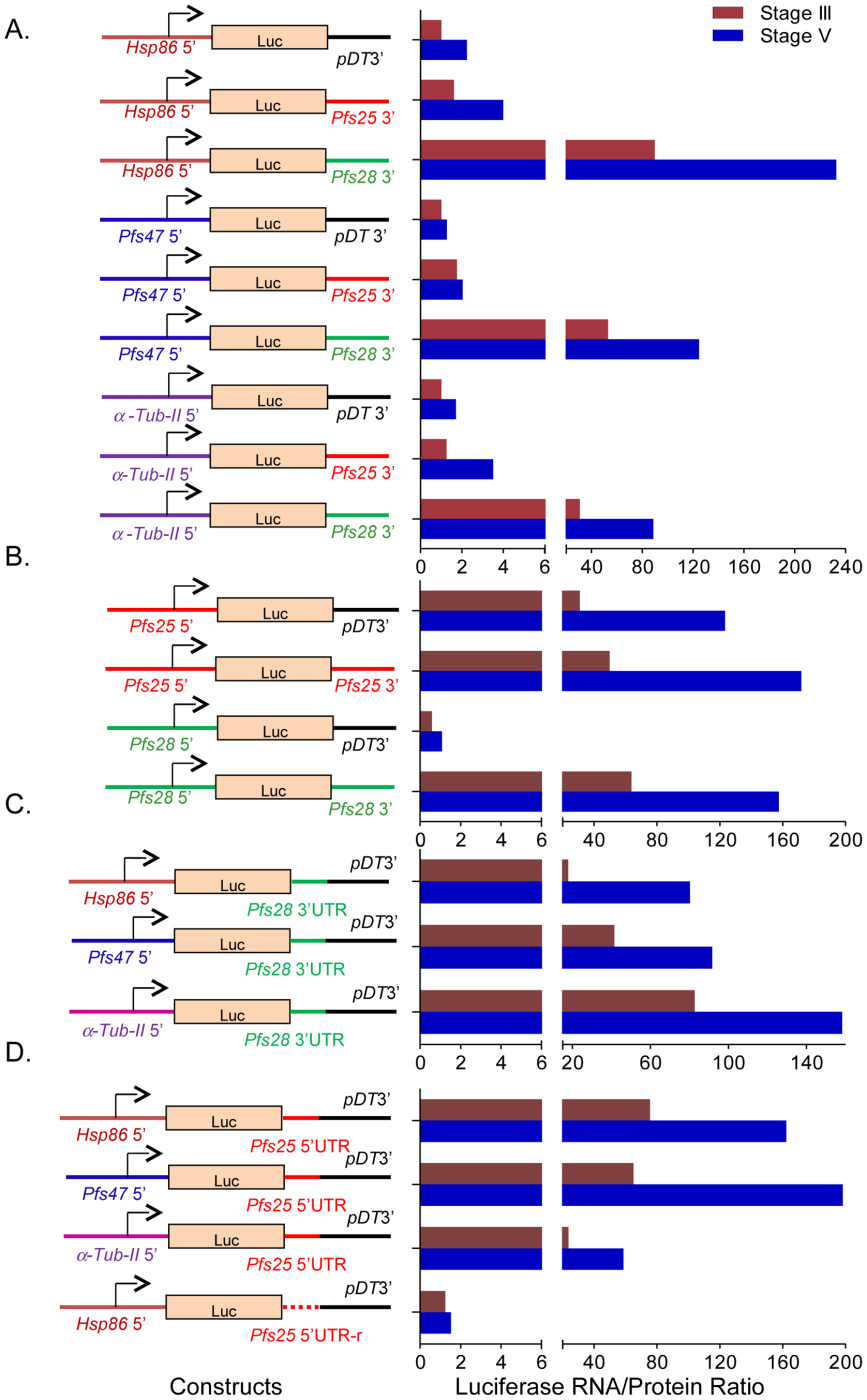
*Pfs28* 3′ and *pfs25* 5′ FRs and UTRs contain *cis*-elements mediating translation repression. Translational repression in gametocytes is mediated by (**A**) *Pfs28* 3′ FR, (**B**) *Pfs25* 5′ FR, (**C**) *Pfs28* 3′UTR, and (**D**) *Pfs25* 5′UTR. Left panel shows schematics of reporter cassettes with *Luc* ORF flanked by the *hsp86*, *pfs47*, or *α-tubulin II* (*α-Tub-II*) promoters, 5′ FRs *pfs25* or *pfs28*, and 3′ FRs of pDT3′, *pfs28, pfs2*5, *pfs28* 3′UTR-pDT3′ or *pfs25* 5′UTR-pDT3′. The dashed line in (D) indicates *pfs25* 5′UTR in reverse orientation (*Pfs25* 5′UTR-r). Right panel shows the corresponding *Luc* mRNA/protein ratios in stage III and V gametocytes of transfected 3D7 parasite line. Luc activities were determined in 100 µg of gametocyte lysates. The relative mRNA abundance was estimated based on real-time RT-PCR analysis using *Luc* primers, and also primers in the UTRs of *pfs25*, *pfs28* and *pDT3*. The positions of the real-time RT-PCR fragments, relative mRNA abundance and Luc activities are shown in Table S2. The relative mRNA levels estimated using the *Luc* primers were used to calculate the *Luc* RNA/protein ratios.

We next focused on the identification of PBEs in *pfs25* and *pfs28*. Because almost all PBEs characterized to date are located in the 3′UTRs of target mRNAs, we first tested the 3′ UTRs of *pfs25* and *pfs28*. Since these genes are female-specific but PfPuf2 is present in both male and female gametocytes, reporter constructs were made with the *firefly luciferase* (*Luc*) gene under the promoters of the constitutively expressed gene *hsp86*, the female-specific gene *pfs47*, or the male-specific gene *α-tubulin II* so that translation regulation can be examined in all gametocytes as well as in a sex-specific manner. The 3′ region of the Luc reporter was derived from ∼1 kb 3′ FR of either *pfs25* or *pfs28*. The 3′ FR of *P. berghei dihydrofolate reductase/thymidylate synthase* (*dhfr/ts*) gene (pDT3′) was included as the non-repressed control UTR [35]. Transfection was done using the site-specific integration system powered by the mycobacteriophage Bxb1 integrase, and the insertion of a single copy Luc expression cassette into the parasite genome was confirmed by integration-specific PCR [46]. The relative abundance of the *Luc* mRNA was evaluated by real-time RT-PCR using a primer pair within the *Luc* ORF and primers in the 3′ region or 5′ region of UTRs (Table S2). The results showed that the relative *Luc* transcript levels in each parasite line assessed, using different sets of primers, were similar. The Luc transcript/enzyme activity ratio in stage III and V gametocytes was used to indicate the translatability of the *Luc* mRNA. Regardless of the promoters used, all constructs with the *pfs28* 3′ FR produced remarkably higher Luc mRNA/protein ratios indicating translational repression, whereas plasmids containing *pfs25* 3′ FR lacked detectable translational repression activity (Figure 5A). To test the possibility of a 5′ FR location of the *pfs25 cis*-regulatory element, four reporter plasmids containing the 5′ FR of either *pfs25* or *pfs28* were evaluated in transfected 3D7 parasites (Figure 5B). Surprisingly, the plasmids containing the *pfs25* 5′ FR displayed evident translation repression activity (Figure 5B).

To further narrow down the locations of the *cis*-regulatory elements in *pfs25* and *pfs28* FRs, constructs with chimeric 3′ FR were made to insert either *pfs25* 5′UTR or *pfs28* 3′UTR (Figure S1) between the *Luc* ORF and pDT3′. Transfection of 3D7 AttB with these *Luc* reporter plasmids containing the UTR-pDT3′ chimeric 3′FR confirmed that both *pfs28* 3′UTR and *pfs25* 5′UTR contained the *cis* elements mediating translation repression (Figure 5C, D). Since the position of translation regulatory elements, such as the iron responsive element in the 5′UTR, is critical for function [47], we tested whether there is a position effect of the *pfs25* 5′UTR for translation repression. Our results showed that the *pfs25* 5′UTR placed in the 3′ end of the reporter construct was mechanistically sufficient for exerting translation repression, but it showed no translation repression activity in the reverse orientation (Figure 5D).

### PfPuf2 is responsible for translational inhibition in an *in vitro* reticulocyte lysate system

**Figure 6:**
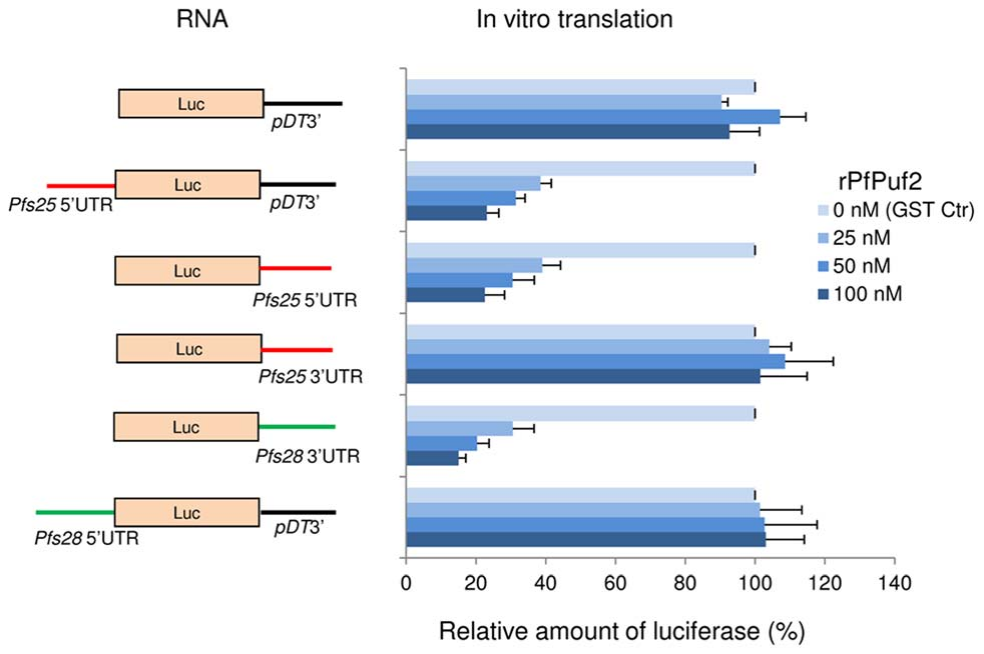
Recombinant PfPuf2 (rPfPuf2) represses translation in an *in vitro* reticulocyte lysate system. Left panel shows schematics of the mRNAs produced by *in vitro* transcription with *Luc* ORF preceded/followed by *pDT*3′, *pfs28* 5′UTR, *pfs28* 3′UTR, *pfs25* 5′UTR or *pfs25* 3′UTR. Equal molar concentrations of the transcripts (2.5 nM) were incubated in rabbit reticulocyte lysates with 25, 50, or 100 nM of GST-rPfPuf2. In the control reactions (GST Ctr), recombinant GST was added at 100 nM. The graph shows the relative amount of Luc (%) produced with regard to the respective mRNAs, which was normalized against the GST control arbitrarily set as 100%. The experiments were done with three replicates and the bars indicate standard deviations.

To corroborate that PfPuf2 is the translation repressor for *pfs25* and *pfs2*8, we employed a cell-free rabbit reticulocyte lysate translation system, where all translational factors are from an evolutionarily distant species. An equal amount (2.5 nM) of the *in vitro* transcribed reporter RNAs was added in each reaction. GST-rPfPuf2 was tested at 25, 50 and 100 nM, which gave a rPfPuf2:reporter RNA ratio of 10∶1, 20∶1 and 40∶1, respectively. As expected, rPfPuf2 at all concentrations tested did not have noticeable effects on translation of the pDT3′ control reporter. Neither was translation repression observed for the reporter mRNAs containing *pfs28* 5′UTR or *pfs25* 3′UTR (Figure 6). In contrast, reactions containing reporter mRNAs with either *pfs28* 3′UTR or *pfs25* 5′UTR at all three GST-rPfPuf2 concentrations produced less than 40% Luc activity of the GST control (Figure 6). Furthermore, *pfs25* 5′UTR positioned either upstream or downstream of *Luc* ORF had a similar translation repression effect on the *Luc* reporter transcript. Since an RNase inhibitor was included in all reactions to prevent degradation of the reporter RNAs, this result also indicated that rPfPuf2 repressed *in vitro* translation of mRNAs containing *pfs25* 5′UTR and *pfs28* 3′UTR.

### *Pfs25* 5′UTR and *pfs28* 3′UTR contain conserved PBEs

**Figure 7:**
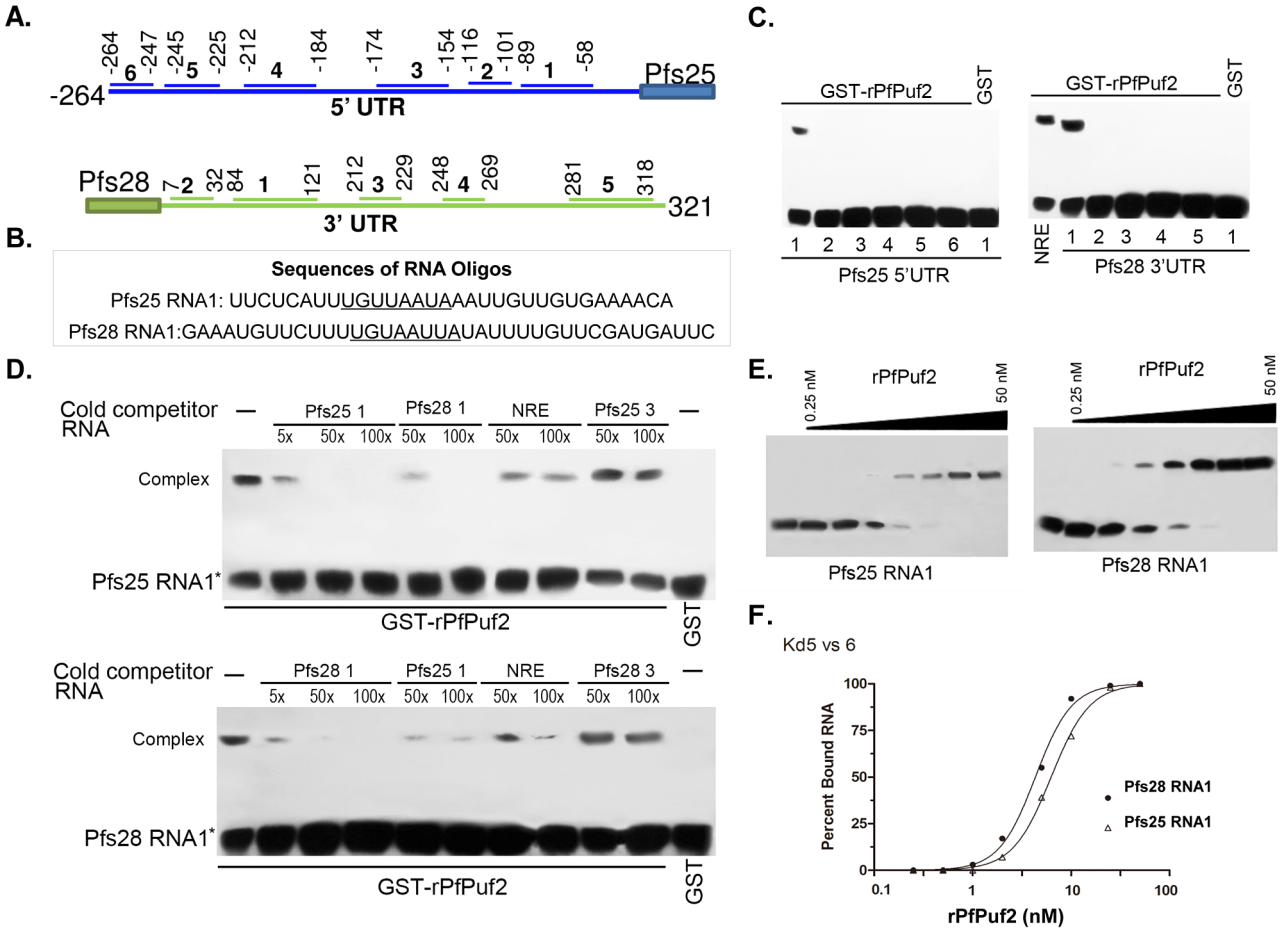
PfPuf2 binds conserved RNA motifs in *pfs25* 5′UTR and *pfs28* 3′UTR. (**A**) Schematics of *pfs25* 5′UTR and *pfs28* 3′UTR showing the positions of the UGU-containing RNA oligos used for EMSA in (C). (**B**) Sequences of two RNA oligos with putative PBE sequences (UGUX_3_UA) underlined. (**C**) EMSA of six RNA oligos from the *pfs25* 5′UTR and five RNA oligos from the *pfs28* 3′UTR (Figure S1). The *Drosophila NRE* sequence was used as a positive control. Lower bands in each gel are the unbound RNA, whereas the upper bands are the protein-RNA complex. (**D**) Competition EMSA showing the specificities of rPfPuf2 binding to *pfs25* RNA1 (upper) and *pfs28* RNA1 (lower). Competitor RNAs were added in reactions at 5×, 50× and 100× of the biotinylated probe (labeled with an asterisk). Either GST-rPfPuf2 or GST was included in the binding assay. (**E**) Representative EMSA images of rPfPuf2 binding*pfs25* RNA1 and *pfs28* RNA1, respectively. Upper and lower bands are protein-RNA complex and unbound RNA, respectively. (**F**) Quantitation of *K*_d_ values based on EMSA analysis from (E). The fraction of RNA bound by rPfPuf2 was plotted versus rPfPuf2 protein concentration. Each value represents the mean from three gels.

Most PBEs studied so far contain a consensus UGUX_3–5_UA sequence [16], [18]–[20], [48]. The *pfs25* 5′UTR and *pfs28* 3′UTR contain 9 and 8 UGU motifs, respectively; each UTR also contains a UGUX_3_UA motif (Figure S1). In addition, the UGUX_3_UA motif was also identified in the *PmVI* 5′UTR and *PFL2320w* 3′UTR (Figure S1). To identify the functional PBEs for PfPuf2, we selected six and five 20–40 bp UGU-containing RNA sequences in the *pfs25* 5′UTR and *pfs28* 3′UTR, respectively, for testing *in vitro* binding to GST-rPfPuf2 using a gel electrophoretic mobility shift assay (EMSA) (Figure 7A, B). To control for non-specific binding, tRNAs (2 µg) were included in each reaction. Consistent with our previous finding, rPfPuf2 bound to the positive control NRE sequence from *Drosophila* [37]. In addition, rPfPuf2 bound to the two UGUX_3_UA motif-containing RNA sequences in *pfs25* or *pfs28*, whereas it did not bind to other UGU-containing RNAs (Figure 7B, C). Similar results were obtained when a poly(U) sequence was used as a non-specific competitor RNA (Figure S3). To further characterize the specificity of the binding, we performed competition experiments, in which unlabeled RNA competitors of *pfs25* RNA1, *pfs28* RNA1, *NRE*, and two negative control RNAs (*pfs25* 3 and *pfs28* 3). Binding to *pfs25* RNA1 and *pfs28* RNA1 was efficiently competed by their cognate RNA and to a lesser degree by the *NRE* RNA, but not by the negative control RNAs (*pfs25* 3 and *pfs28* 3) (Figure 7D). In addition, effective cross competition was also observed between *pfs25* RNA1 and *pfs28* RNA1. These results indicate that the binding of rPfPuf2 to *pfs25* RNA1 and *pfs28* RNA1 is specific. Quantitation of the binding affinities showed that rPfPuf2 binds to *pfs25* and *pfs28* RNA1s with *K*_d_ values of 11.5±3.8 and 6.7± 2.5 nM, respectively (Figure 7E, F).

### The PBEs of *pfs25* 5′UTR and *pfs28* 3′UTR are functional

**Figure 8:**
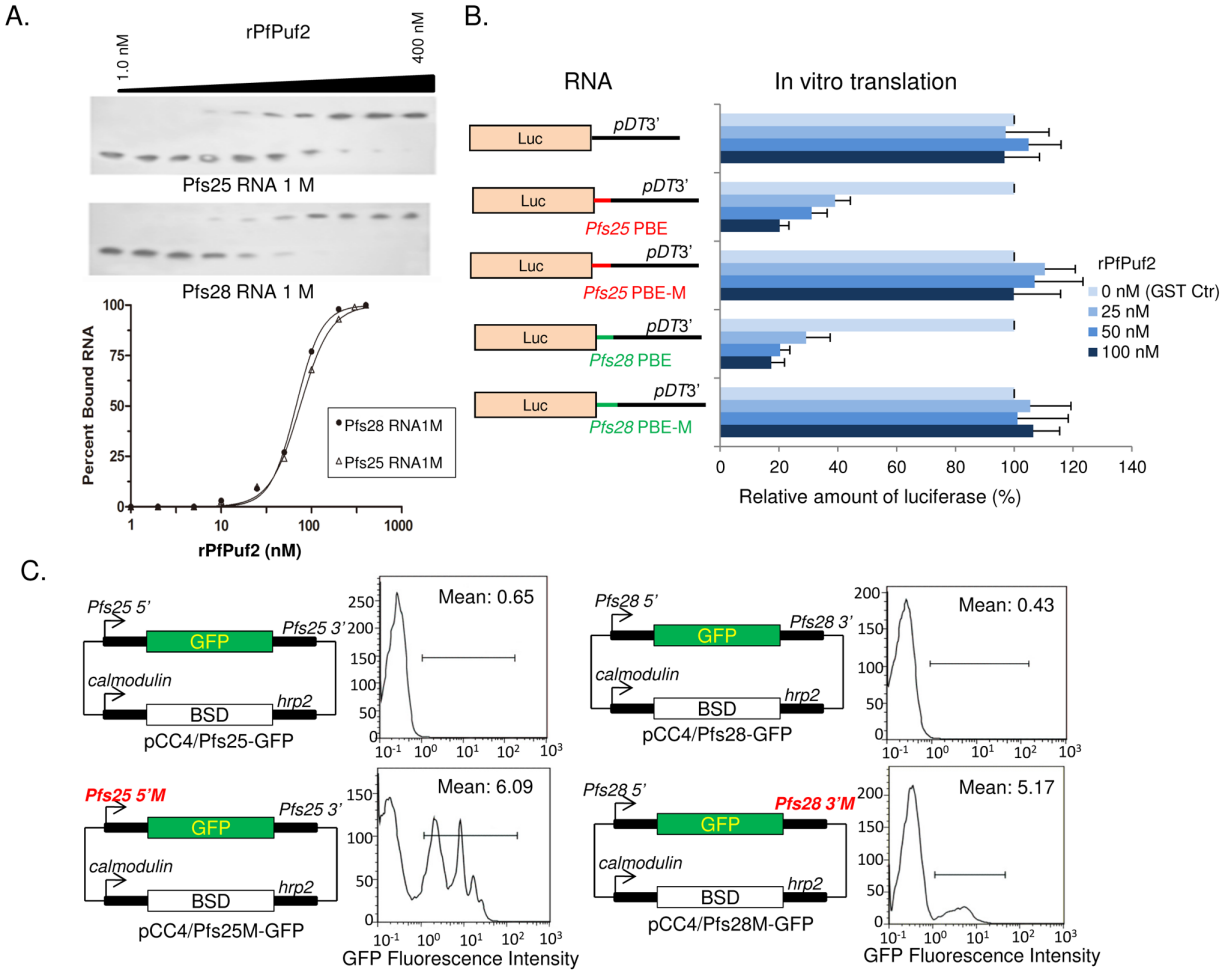
Mutation analysis of the putative PBEs in *pfs25* 5′UTR and *pfs28* 3′UTR. (**A**) Mutation of the UGU sequence to UCC in the PBEs of *pfs25* RNA1 and *pfs28* RNA1 reduces the rPfPuf2 binding affinity. *K*_d_ values were quantified based on EMSA analysis of the mutant RNAs. Upper panel shows a representative EMSA gel picture, and the lower panel shows the sigmoid curve for the estimation of *K*_d_ based on average values from three gels. (**B**) The UGU to UCC mutations in the PBEs abolish inhibition of protein translation in the cell-free system. Left schemes show the mRNAs containing *Luc* ORF (Luc) followed by pDT3′ (as the control), as well as with the insertion of the wild-type *pfs25* RNA1 (*pfs25* PBE) and *pfs28* RNA1 (*pfs28* PBE) and their respective mutant sequences *pfs*25 PBE-M and *pfs28* PBE-M. Right panel shows the relative amount of Luc (%) produced with regard to the respective mRNAs, which was normalized against the translation control (no rPfPuf2, 100 nM GST) arbitrarily set as 100%. (**C**) The UGU to UCC mutations in the PBEs of *pfs25* 5′UTR and *pfs28* 3′UTR led to a loss of translation control *in vivo* in 3D7 parasites. Left panels show schematics of the plasmids with the wild-type UTRs and mutant UTRs with the UGU to UCC mutations (highlighted in red). Transfected parasites were selected under 30 ng/ml of blasticidin. Right panels show quantitation of GFP-positive gametocytes by flow cytometry as described in Figure 4C.

For further validation of the PBEs, mutation analysis was performed and evaluated both *in vitro* and *in vivo*. Since the UGU sequence within the PBEs is the most conserved and essential for Puf binding [48], we mutated the UGU in the UGUX_3_UA motifs to UCC and tested whether the mutations affected interactions with PfPuf2. In EMSA, the binding affinities of the mutant RNAs to rPfPuf2 were significantly reduced (Figure 8A) with *K*_d_ values for *pfs25* RNA1M and *pfs28* RNA1M increased to 169.3±56.8 and 142.8±54.9 nM (∼15- and ∼21-fold reductions in affinity), respectively. To test the effect of the mutations on translation repression by rPfPuf2 in the cell-free reticulocyte lysate translation system, reporter RNAs were made to contain chimeric 3′UTR where either wild-type or mutant PBEs from pfs25 RNA1 and pfs28 RNA1 were inserted between the *Luc* ORF and pDT3 UTR (Figure 8B). *In vitro* translation was performed and showed that rPfPuf2 lost translation inhibition of the *Luc* mRNAs containing the UCC mutation (Figure 8B). To verify the functionality of the two PBEs *in vivo*, the UGU sequence in the UGUX_3_UA motif was mutated to UCC in the GFP reporter plasmids shown in Figure 4. Constructs carrying both wild-type and mutant motifs were transfected into 3D7 and parasites were selected with 30 ng/ml of blasticidin. Flow cytometry analysis of purified gametocytes showed that parasites carrying the mutant PBEs displayed significantly increased mean GFP signal intensities (Figure 8C). Besides, increasing the drug selection pressure to 240 ng/ml blasticidin did not result in significant changes in GFP signal intensity in gametocytes, indicating that the UCC mutation resulted in a loss of translational repression.

## Discussion

This study demonstrates an important role of a Puf family protein in regulating protein translation during developmental transitions in *P. falciparum*. Whereas studies carried out to date support the paradigm of Puf-mediated translational regulation through PBEs in 3′UTRs, our study provides both *in vitro* and *in vivo* evidence for the 5′UTR localization of a functional PBE. This finding demonstrates the flexibility of Puf proteins in selecting PBEs within target transcripts. The identification of many TR transcripts in *P. falciparum* gametocytes as potential target genes suggests that PfPuf2 is an important translational regulator during the developmental transition from humans to mosquitoes.

### Puf2 represses translation of target mRNAs

To date, most known Puf proteins are translational repressors, and they bind target mRNAs to affect their stability, localization and translation [8]. For example, deletion of Puf3 in yeast results in a selective increase in relative abundance of many Puf3-associated mRNAs [13]. Specifically, the *HXK1* mRNA becomes more stable and Hxk1p is more abundant when Puf1, Puf4 or Puf5 is disrupted [49]. Likewise, expression of FOG-2 protein in *C. elegans* is increased in the *fbf-1/puf-8* double mutant [50]. If PfPuf2 affects the stability of its mRNA targets, disruption of Pufs would lead to enhanced stability and increased abundance of the target mRNAs, as well as de-repression of their translation [5], [8]. At least for Pfs25, Pfs28 and PmVI, PfPuf2 deletion led to increased mRNA and protein levels in gametocytes, whereas overexpression of PfPuf2 further reduced their mRNA and protein levels. Based on this result, we speculate that PfPuf2 might be associated with accelerated decay of these target mRNAs in *P. falciparum* gametocytes. Furthermore, translation repression of reporter RNAs by rPfPuf2 in a reticulocyte lysate system where RNA degradation was prevented suggests that rPfPuf2-binding to target mRNAs may directly inhibit their translation or through interactions with regulatory factors present in the lysates. Although it is possible that the two potential mechanisms might be acting in concert in PfPuf2-mediated translational repression, these data indicate that translation repression in *P. falciparum* gametocytes is not complete. In contrast, the single DOZI system in *P. berghei* appears to be fully effective [33], [34], which further highlights the dramatic differences in gametocyte biology between *P. falciparum* and *P. berghei* [51]. The leaky expression of TR transcripts in *P. falciparum* gametocytes also implies that these proteins may elicit antibody responses in people living in malaria hyperendemic areas after repeated exposure to malaria infections, and therefore may serve as potential markers for immune detection of gametocytes.

Although the exact mechanism remains to be elucidated, Puf family proteins are found to affect mRNA stability by interacting with and recruiting additional factors. Of particular interest is that Puf proteins in several organisms interact with Pop2, a key component of the Ccr4-Pop2-Not deadenylation complex [8], thereby recruiting deadenylase to mRNAs. Interestingly, this complex also interacts with the decapping activator and translational repressor Dhh1, a DEAD-box RNA helicase, which affects the integrity of the 5′ cap structure and leads to translation repression [52]. Intriguingly, in the gametocytes of *P. berghei*, DOZ1, the Dhh1 homolog, protects TR transcripts including the orthologs of *pfs25* and *pfs28* from degradation by forming mRNA nucleoprotein particles reminiscent of “P-granules” [33]. However, the DOZI complex in *P. berghei* gametocytes does not contain the Puf proteins [34], suggesting DOZI and Puf2 are different protein complexes. In addition, there are very limited overlaps between up-regulated transcripts in PfPuf2 deletion and down-regulated genes in *pbdozi* and *pbcith* deletions, indicating that DOZI and Pufs function in different pathways even though they potentially act on a similar set of TR transcripts.

### Pufs mediate translation regulation via conserved PBEs in both 5′ and 3′UTRs

All target genes of Puf family proteins characterized so far contain *cis*-regulatory PBEs located in the 3′UTRs and 3′ UTR proximal ORF [53], which leads to the generalization of Puf-mediated translational regulation through 3′UTRs [5], [8]. The mechanism of 3′UTR-mediated translation regulation by Pufs is explained by the closed-loop model of mRNAs [5], [8], [54], [55]. In this study, by comparatively transfecting 3D7 and ΔPuf2 with reporter constructs, we found that the translation repression of four selected genes *pfs25*, *pfs28*, *PFL2320w* and *PmVI* was mediated by *cis*-regulatory elements located in their UTRs and that this regulation was PfPuf2-dependent. A focused analysis of *pfs25* and *pfs28* allowed us to further narrow down the locations of the *cis*-regulatory elements to *pfs28* 3′UTR and *pfs25* 5′UTR. These results demonstrate that PfPuf2 is a versatile translation regulator and acts through both 3′ and 5′ UTRs. Interestingly, two UGUX_3_UA elements are also found in the *PmVI* 5′UTR, and one of them is identical to the functional *pfs28* PBE motif (Figure S1), suggesting that translation regulation of *PmVI* might also be via the 5′UTR. Since the only confirmed functional PBE is distal to the 5′ end of the *pfs25* mRNA, we can envisage that binding of PfPuf2 to this site may inhibit scanning of the 40S ribosomal subunit. In addition, the 5′UTR element from *pfs25* also functions in the 3′UTR position, suggesting that a similar regulation mechanism might be used for both 5′ and 3′UTRs.

The discovery of a PBE in the 5′UTR raises a question about whether this phenomenon is unique in *Plasmodium* or common among other organisms. Interestingly, the 5′ UTR of *pbs21* (ortholog of *pfs25*) also confers translational repression in *P. berghei* gametocytes through a 47-base U-rich, *cis*-acting, element [35], but similar sequences are not present in *P. falciparum*. Genome-wide analyses for Puf-associated mRNAs performed in model organisms identify 3′UTR locations of most putative PBEs, but smaller proportions of these mRNAs contain putative PBEs in the 5′UTRs and ORFs [16], [18]. So far, only one PBE localized in the 3′UTR-proximal ORF in the *para* gene of *Drosophila* neurons has been confirmed to be the target of Pumilio [53], whereas none of the putative 5′UTR PBEs have been validated. Therefore, it would be interesting to see whether Puf-mediated regulation through 5′UTR can be expanded to other organisms. In mRNA targets of Pufs in yeast, humans and *Drosophila*, the most commonly found PBEs contain a “UGUX_3–5_UA” sequence motif [16], [18]–[20], [48]. In our study we found that PfPuf2 binds to the single “UGUX_3_UA” motif present in either *pfs25* 5′UTR or *pfs28* 3′UTR and these canonical PBEs are functional *in vivo*, suggesting that the molecular mechanism of Puf-mediated translation regulation might be conserved in this lower branch of eukaryotes.

### Puf2 is a regulator of translation in *Plasmodium* gametocytes

Based solely on the abundance of PBE-like motifs in the AT-rich *P. falciparum* genome, it is difficult to predict the exact repertoire of the target genes regulated by PfPuf proteins. Judging from the changes in the mRNA levels in ΔPuf2 parasites at gametocyte and sporozoite stages [39], and the relative abundance of Puf-associated mRNAs in model organisms, Puf2 may be a regulator of a large number of target genes. Further, the expression of Puf2 in both male and female gametocytes suggests that Puf2 regulates target gene expression in both sexes [40]. Indeed, our microarray data showed that disruption of PfPuf2 caused changes in the steady-state mRNA level of ∼13% of genes in stage III and V gametocytes, suggesting that PfPuf2 may potentially regulate a large set of transcripts. It is noteworthy that PfPuf1 is also abundantly expressed in gametocytes and it may be another regulator of translation. Further studies of the mechanisms of translation regulation by Puf proteins and characterization of the target mRNA repertoires will help better understand the biology of sexual development in malaria parasites.

## Materials and Methods

### Parasite culture and gametocytogenesis

Cultures of *P. falciparum* 3D7, ΔPuf2, and PfPuf2 over-expression clones in 3D7 and ΔPuf2 were maintained as described previously [56]. A modified *in vitro* induction scheme for gametocytogenesis was used to obtain highly synchronous gametocyte cultures [57].

### Microarray analysis

For microarray analysis of gene expression in gametocytes between 3D7 and ΔPuf2, a custom-designed expression microarray was designed by Roche NimbleGen (Madison, WI) based on the *P. falciparum* 3D7 genomic sequence [36]. Total RNA was extracted with Trizol Reagent from 1×10^7^ purified stage III and stage V gametocytes, respectively. RNA was amplified and labeled with Cy5 or Cy3 using Amino Allyl MessageAmp II aRNA Amplification kit (Ambion, Austin). RNA labeling, array hybridization, and signal normalization were done as described [36]. The experiment was replicated three times. Microarray data were analyzed by *t*-statistics, the SAM method [42], and comparison of fold changes of average transcript levels. Microarray data were submitted to NCBI GEO with an accession number of GSE39124.

### DNA reporter constructs

To episomally express GFP under the control of the FRs of *pfs25*, *pfs28*, *PmVI*, *PFL2320w* and *α-tubulin II*, ∼1 kb fragments of the 5′ and 3′ FRs of these genes were PCR amplified from *P. falciparum* genomic DNA using primers listed in Table S3 and cloned to flank the *gfp* ORF in the pCC4 vector [58] to obtain plasmid spCC4/Pfs25-GFP, pCC4/Pfs28-GFP, pCC4/PmVI-GFP, pCC4/PFL2320w-GFP, and pCC4/tubulin-GFP, which all contain the drug selection cassette with the blasticidin S deaminase (BSD) ORF flanked by the *calmodulin* promoter and *hrp2* 3′ region (Figure 4).

To study the effects of 5′ and 3′UTRs on translational regulation, a site specific integration system was used to insert a single copy of the Luc expression cassette into the parasite genome [46]. For expression of Luc in different sexes of gametocytes, *Luc* ORF was flanked by a ∼1 kb fragment from the 5′ FR of either *pfs47*, *α-tubulin II*, *pfs25*, *pfs28*, or an 800 bp fragment from the 5′ FR of *hsp86* and ∼1 kb fragment from the 3′ FR of either *pfs25, pfs28* or pDT3′ (Figure 5, Table S3). To narrow down the locations of the *cis* elements, the *pfs28* 3′ UTR and *pfs25* 5′UTR were amplified and inserted between the Luc ORF and pDT3′ (Figure 5, Table S3). A construct with the *pfs25* 5′UTR inserted in the reverse orientation was also included in the analysis. These Luc expression cassettes were cloned into the pLN-NRE-GFP vector to replace the GFP expression cassette [46].

### Parasite transfection

Parasite transfection was performed as described earlier [40]. For episomal expression, transfected parasites were selected under 30 ng/ml of blasticidin with weekly replenishment of RBCs until resistant parasites were observed. To increase the copy number of the episomal plasmids and expression of the GFP reporter, parasites were cultured under 240 ng/ml of blasticidin. For the site-specific integration system, 3D7 AttB parasites were co-transfected with the pLN constructs (containing the Luc expression cassettes) and pINT (encoding the integrase) and selected with 2.5 nM WR99210, 2.5 µg/ml blasticidin and 125 mg/ml G418 [46]. Integration-specific PCR was performed using primers in Table S3 to confirm integration of the Luc expression cassette at the *cg6* locus in the parasite genome [46].

### Determination of reporter expression

Reporter (*gfp*, *Luc*) mRNA levels in gametocytes were quantified by real-time PCR using at least two sets of primers (Table S2, 3). The relative mRNA level of each gene was calculated using the ΔC_t_ method using *PF07_0073* as the internal reference [59]. GFP expression in parasites was observed under a fluorescence microscope [40]. Stage III and V gametocytes were purified by centrifugation on a 35%/70% Percoll step gradient (v/v) [60]. GFP fluorescence intensity in gametocytes was measured using the Coulter XL-MCL Flow Cytometer and mean fluorescence intensity was calculated. Excitation was performed with a 488 nm argon-ion laser and GFP was detected using a 525 nm band pass filter. Luc activity in gametocyte lysates was determined using the Luciferase Assay System (Promega). The Luc mRNA/enzyme ratios were used to show translatability of the mRNAs with higher ratios indicating translation inhibition. In parasites transfected with the constructs containing *hsp86*, *pfs47* or *α-tubulin II* 5′ FR and the control pDT3′, the Luc mRNA/protein ratio was arbitrarily set as 1.

### RACE

To determine 5′ and 3′UTRs of *pfs25*, *pfs28*, *PmVI*, and *PFL2320w*, total RNA was isolated from mature gametocytes. The transcription start sites were determined using the FirstChoice RNA-ligase mediated-RACE Kit (Ambion, Austin, TX) as described before with gene-specific outer and inner primers (Table S3) [37]. The 3′ polyadenylation sites were determined by 3′RACE using oligo-dT primer and gene-specific primers (Table S3).

### Site directed mutagenesis

The PBEs in *pfs25* 5′ and *pfs28* 3′ UTRs within the plasmids pCC4/Pfs25-GFP and pCC4/Pfs28-GFP were mutated by QuikChange II XL Site-Directed Mutagenesis Kit (Agilent Technologies, CA) from TGT to TCC to generate plasmids pCC4/Pfs25M-GFP and pCC4/Pfs28M-GFP, respectively (Figure 8).

### *In vitro* inhibition of translation

To verify that PfPuf2 mediates translation repression of target genes, we used a cell-free translation assay [61], which included rPfPuf2 and different mRNA species in the Flexi Rabbit Reticulocyte Lysate System (Promega). The rPfPuf2 was expressed and purified as a GST-fusion protein [37], and added in the *in vitro* translation system at 0, 25, 50, and 100 nM. In the control, recombinant GST was added at 100 nM. For reporter mRNAs, *Luc* preceded by *pfs25* 5′UTR and *pfs28* 5′UTR or followed by pDT3′, *pfs25* 5′UTR, *pfs25* 3′UTR, or *pfs28* 3′UTR was PCR amplified (Figure 6). A T7 promoter sequence and a 50-nucleotide poly(A) tail were added to the 5′ and 3′ ends of the PCR products, respectively, using additional primers (Table S3). In addition, wild-type *pfs25* RNA1 and *pfs28* RNA1 and their reverse sequences as well as primers containing the TGT to TCC mutations were synthesized, annealed and inserted between the Luc ORF and pDT3′ (Figure 8 and Table S3). All PCR products were finally prepared for *in vitro* transcription using the T7 RNA polymerase in the MEGAscript kit (Ambion, Austin). *In vitro* translation was performed in triplicate in 50 µl at 30°C for 60 min with 2.5 nM of each reporter RNA and Luc activity was quantified using the Luciferase Assay System. To inhibit potential RNA degradation, RNase inhibitor was included in the reactions at 50 units/reaction. The amount of Luc produced in the GST control was designated as 100%.

### *In vitro* RNA binding assay

EMSA was performed using the LightShift Chemiluminescent RNA EMSA kit (Pierce, Rockford). Briefly, each 20 µl of reaction contained 2 µg tRNA for blocking non-specific RNA-protein interactions, EMSA binding buffer, 20 units of RNase inhibitor, 5% glycerol, rPfPuf2 (or GST control), and biotinylated RNA oligos with or without cold competitors. The *Drosophila NRE* RNA sequence (Table S3) and a total of 11 UGU-containing RNA sequences in the *pfs25* 5′ UTR and *pfs28* 3′ UTR (Figure S1), and Pfs25 1M and Pfs28 1M (Mutation from UGU to UCC) were synthesized as biotin-labeled RNA fragments (Integrated DNA Technologies). In a 20 µl reaction, 2.5 nM of an RNA oligo and different concentrations of rPfPuf2 (from 0.25 to 400 nM) were incubated at room temperature for 20 min. Cold competitor RNAs were included at 5×, 50× and 100× concentrations of the biotinylated RNAs. The reactions were electrophoresed on a 5% native acrylamide/8M urea gel and transferred to a nylon membrane. The bands of labeled oligos were detected using the Chemilumescent Nucleic Acid Detection Module (Pierce). Each experiment was repeated three times and the average *K*_d_ values were estimated by fitting the curves to the mean percentages of the total bound RNA, which were determined by densitometry using the Quantity One 1-D Analysis Software (BioRad, Hercules, CA).

### IP and quantification of associated mRNAs

IPs were performed using the parasite line carrying GFP-tagged PfPuf2 [40]. Mature gametocytes were purified by Percoll gradient centrifugation and lysed in 5 volumes of the PA150 buffer (150 mM KCl, 20 mM Tris-HCl, pH 7.7, 3 mM MgCl_2_, 0.5 mM DTT, 0.1% Tween 20) containing a protease inhibitor cocktail (Roche) and 2 units/µl of RNasin (Promega). The lysate was centrifuged for 10 min at 20,000× g and the supernatant was incubated with 5 µg of monoclonal anti-GFP antibody (Roche) or 10 µg of anti-c-Myc antibody (Sigma) for 1 h on ice with occasional mixing. Protein A agarose (30 µl packed bead volume) was added to the lysate and incubated for 1 h. The agarose beads were washed four times with PA150 and divided in two equal volumes: one was re-suspended in SDS-PAGE loading buffer for immunoblotting analysis and another was used for RNA extraction using TRIzol Reagent. Extracted RNA was used for cDNA synthesis and real-time RT-PCR as previously described [59] with primers designed for putative TR transcripts (*pfs25*, *pfs28*, *pfL2320w*, *pfc0495w*, and *Pf14_0508*) and an internal reference gene *PF07_0073* (Table S3).

### Immunoblotting

To compare expression of Pfs25, Pfs28 and PmVI in 3D7 and ΔPuf2 gametocytes, proteins from equal numbers of mature gametocytes (10^6^) were separated in a 12% SDS-PAGE gel. Proteins were transferred to a nitrocellulose membrane and probed with primary antibodies against these three proteins at 1∶1000 and secondary horseradish peroxidase-conjugated antibodies at 1∶3000. Proteins were visualized using an ECL Kit (Invitrogen). The rabbit anti-PmVI antibodies were generated by immunization with purified recombinant PmVI protein expressed in *E. coli*. Monoclonal antibodies against Pfs25 and Pfs28 were obtained from MR4. For detecting PfPuf2-GFP after IP, anti-GFP antibodies (Roche) at 1∶1000 were used.

### Statistical analysis

To compare GFP RNA levels and fluorescence intensity between transfected 3D7 and ΔPuf2, the data were log-transformed and *t*-tests were performed with a Bonferroni correction.

## Supporting Information

Figure S1 **The major initiation and termination sites of** ***pfs25*** **,** ***pfs28*** **,** ***PmVI*** **and** ***PFS2320w*** **.** These sites (shown in bold) are determined by rapid amplification of cDNA ends (RACEs). Six and five RNA oligos selected for electrophoretic mobility shift assay (EMSA) in *pfs25* 5′ UTR and *pfs28* 3′ UTR, respectively, are underlined. The UGU trinucleotides were shaded, and the 8-nucleotide motifs resembling the Puf binding element core consensus (UGUX_3–5_UA) are highlighted in red. Note that the UGUX_3_UA motif is also found in the *PmVI* 5′ UTR and *PFL2320w* 3′UTR.
(DOCX)

Figure S2 **Transcription of** ***gfp*** **in wild-type 3D7 and ΔPuf2 lines.** These lines were transfected with the GFP expression cassette under the control of *pfs25* or *pfs28* FRs. Gametocytes at stage III (day 8) and stage V (day 12) were harvested for RNA purification. The relative GFP RNA levels were measured by real-time RT-PCR using *PF07_0073* as a reference. RNA levels were significantly increased when the drug pressure was elevated to 240 ng/ml of blasticidin (*P*<0.001, *t*-test).
(PDF)

Figure S3 **Recombinant PfPuf2 binds to** ***pfs25*** **5′UTR and** ***pfs28*** **3′UTR in the presence of poly(U) as a competitor.** ESMA of six RNA oligos from the *pfs25* 5′UTR and five RNA oligos from the *pfs28* 3′UTR (Figure S1) using the same conditions as in Figure 7 except that 100 nM of poly(U) was included in each reaction as a non-specific competitor. The *Drosophila NRE* sequence was used as a positive control. Lower bands in each gel are the unbound RNA, whereas the upper bands are the protein-RNA complex.
(PDF)

Table S1 **Results from microarray analysis.** (**A**) Up- and down-regulated transcripts in stage III gametocytes between 3D7 and ΔPfPuf2 line. (**B**) Up- and down-regulated transcripts in stage V gametocyte between 3D7 and ΔPfPuf2 line. (**C**) Up- and -down-regulated transcripts in both stage III and stage V gametocyte between 3D7 wild type and ΔPfPuf2 line. (**D**) Shared transcripts between up-regulated (>2 fold) in ΔPfPuf2 gametocytes (Stage III and V) and down-regulated in both ΔDOZ1 and ΔCITH in P. berghei gametocytes.
(PDF)

Table S2 **Relative** ***luciferase*** **mRNA levels and luciferase activities in gametocytes of stably transfected 3D7-AttB parasites.**
(PDF)

Table S3 **Primers for PCR, mutagenesis, RACE, and** ***in vitro*** **transcription.**
(DOCX)
