# Supplementary material for: Puf Mediates Translation Repression of Transmission-Blocking Vaccine Candidates in Malaria Parasites
Source: PLoS Pathog. 2013 Apr 18;9(4):e1003268. doi: 10.1371/journal.ppat.1003268 (PMC3630172; doi:10.1371/journal.ppat.1003268)
Supplement: Figure S1 — The major initiation and termination sites of pfs25 , pfs28 , PmVI and PFS2320w . These sites (shown in bold) are determined by rapid amplification of cDNA ends (RACEs). Six and five RNA oligos selected for electrophoretic mobility shift assay (EMSA) in pfs25 5′ UTR and pfs28 3′ UTR, respectively, are underlined. The UGU trinucleotides were shaded, and the 8-nucleotide motifs resembling the Puf binding element core consensus (UGUX3–5UA) are highlighted in red. Note that the UGUX3UA motif is also found in the PmVI 5′ UTR and PFL2320w 3′UTR. (DOCX) [file ppat.1003268.s001.docx]

**Pfs25**

**5’UTR**

**A**UUUUGUUAAUUUAUAAAAUAUU**A**UUAUGUCGUUUUUAUAAACUUCAAUAUAGAUUUUUAUUUGUAAUUGUUUUUAUUAGAAUCUUAUUU

6 5 4

UAUUUCUUUGUUUUCUUCAAUUUAUUCAUUUUUUUCGUUAUCUUUUUUUUUUUUUUUUUUUUGUCUUAUUAUUUUUAUUUUAUUUUUCUC 3 2

AUUUGUUAAUAAAUUGUUGUGAAAACAAAAAAACAAAAAAAAACAAAAAAAACUCAUACCUUAUAUUUUUUUAUUCUUUUAAAA

1

**3’UTR**

ATATTATAACAACATATATATATATTTTAAATGGTAAATTATCAAGGTTAAAACACTTTTTACAGTTATGAATAATTAATTGAACTTTTATATGTATTATTATTATTATTATTACTATTGTTATTTTTGGTTATTCTTTTTTATTACTATTATTTATTTTTTTTTATATTTTTTTTTTTT

TTTTTTATATTTATTTATTTTTTTTTTTTTTTTTTTTTTTTTTTATTAATTGCTATAAAGTGGATAATTATTTATTATAATATAATAAATATATTTATAGATCAAATTATATTTAAGTTTTCTCTGAACATTGAAAAATTATAATTTCTTATAAGACAAAAAAAAATACCATGAACTCTA

ATAAAATGATATCTA**A**TATTAAAATTTGGTAAATTCAGAA**A**

**Pfs28**

**5’UTR**

**A**TATATTCATTAAAAATAAAATTAAAATAAATAAATAACGATCTATACTTATATGTACAAAAGAACAAAACTTATAAGAATAAATATAAAT

TATATATACTCACAAAATATTGTTATAAATCTTACTTATTTGTGTTTCTTTTTTTATGGTCAATTTGTCTTACATATACATGAATATTGTGCACAAAATTATATAAATTGTAATCTTTTTTTTTTTTTTCTTTTTTTTTTTAAATTTTATATATTTATAGATATGTACATTATTAATAATCACTTATTATTTTATATTTTTTAGTTTTTTATACA

**3’UTR**

AAAAAAAAUAUAUAUAUAUGUAUAUAUAUAUAUAUAUAUAUAUAUAUAUAUAUAUAGUCAUAUGAUUUGCAUCUUAUUUCUUUGAAAUGU

2

UCUUUUGUAAUUAUAUUUUGUUCGAUGAUUCAACAAAUUAUAUAUGACAUAUUAUACAUUAUAUAAAAAAAAUAAAAUAAAAUAAAAUAA

1

AAUAGAUAUUAUAUAUAUAUAUAUAUAUAUAUAUAUAUGUGCUUCUUUUUUUUUUUUUUUUUAUACCGUUUAAUUGUUUAAACCUAACUA

3 4

AUAAAACACAUUUUAUAAGUUGUGCAAAAA**A**CAUAUUGUUUUCUUAAAAA**A**

5

**PmVI**

**5’UTR**

UCA**U**UUUU**A**UUUCAUCAUUUAACAUAUAGUCUUAUUUUUUAUUAUUUUUUAUUUUUUUGUCCAUUUAAACAUUCAUCAUAAAAACUUAUAAAUUAUCUACAAAAUAUAUUUACUUGUUUUUAUUCCAGAAAUUUUAUAUGUAAUUAAAAAAAAAAAAAAAAAAAAAAAAAAAA

**3’UTR**

AAAACAAAAAAAAGUGCAGGAUUAUUGUAACACAUAUGA**A**UAUAUUCAUAAUAUUUUUUUAUUUUAUUUAUUUUUUUUUUUAUUUAUUUUGUUUUUGUUUUUUUUUUUUUUUUUUUUUUUUUUUGAAUUGGGUAAAAACUUAACAUACAAUAUAUUUAUAUAUAUAUAUAUAUAUAAUAUGUAACACAUCAAUUAUUUAUUUUAUUUUUUUAAGGUAUUUUUUUUUUUUUUUUUUUUUUUCUUUUU**A**AA**A**

**PFL2320w**

**5’UTR**

UUUUUUAU**A**UU**G**UUUAAUUUAACUAGCCAUUUUGUUGUAUUAUUAUAAUUUUAACAUAAUUAGAAAAAUAACAUAAGUAUAAAAUAUAUACAUAUAUAUAUAUUUUUUAUUUAUAUAUCUAAAAUUUUCAGCUUUUAAGAAUG

**3’UTR**

AUGAAUAUAUACAUAUAUAUAGAUAUAUAUAUAUAGAUAUAUAUAGGUAUAUAUUUACUUUAAAAUUCCUUAAAUGAGUAAAGGAAAAAACAAAGUAGUCUAUUUUUUUUUUUUUUUUUUUUUUUUCAAAUGUUUUCAACAAUAAAAUGUAUAUAUAUAUAUAUAUAUAUAUAUAAAUAUAUAUUUAUACAUAUGUGCU**A**A**A**

**Figure S1. The major initiation and termination sites of *pfs25*, *pfs28*, *PmVI* and *PFS2320w***. These sites (shown in bold) are determined by rapid amplification of cDNA ends (RACEs). Six and five RNA oligos selected for electrophoretic mobility shift assay (EMSA) in *pfs25* 5' UTR and *pfs28* 3' UTR, respectively, are underlined. The UGU trinucleotides were shaded, and the 8-nucleotide motifs resembling the Puf binding element core consensus (UGUX_3-5_UA) are highlighted in red. Note that the UGUX_3_UA motif is also found in the *PmVI* 5' UTR and *PFL2320w* 3'UTR.
