# Supplementary material for: Puf Mediates Translation Repression of Transmission-Blocking Vaccine Candidates in Malaria Parasites
Source: PLoS Pathog. 2013 Apr 18;9(4):e1003268. doi: 10.1371/journal.ppat.1003268 (PMC3630172; doi:10.1371/journal.ppat.1003268)
Supplement: Figure S2 — Transcription of gfp in wild-type 3D7 and ΔPuf2 lines. These lines were transfected with the GFP expression cassette under the control of pfs25 or pfs28 FRs. Gametocytes at stage III (day 8) and stage V (day 12) were harvested for RNA purification. The relative GFP RNA levels were measured by real-time RT-PCR using PF07_0073 as a reference. RNA levels were significantly increased when the drug pressure was elevated to 240 ng/ml of blasticidin (P<0.001, t-test). (PDF) [file ppat.1003268.s002.pdf]

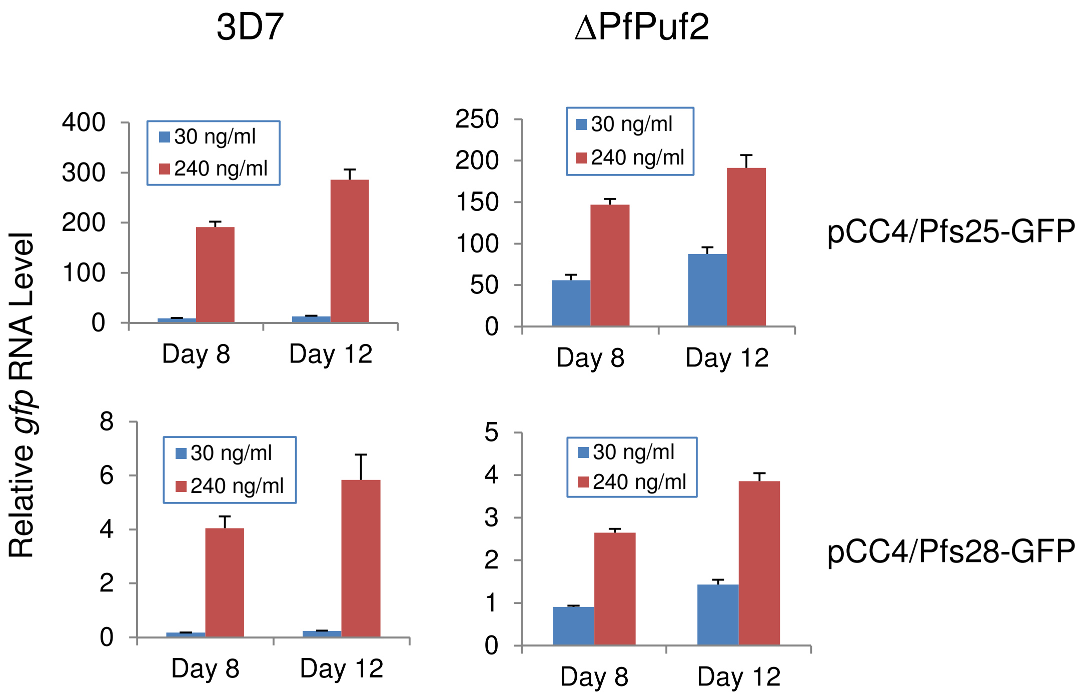


**Figure S2. Transcription of *gfp* in wild-type 3D7 and ΔPuf2 lines**. These lines were transfected with the GFP expression cassette under the control of *pfs25* or *pfs28* FRs. Gametocytes at stage III (day 8) and stage V (day 12) were harvested for RNA purification. The relative GFP RNA levels were measured by real-time RT-PCR using *PF07_0073* as a reference. RNA levels were significantly increased when the drug pressure was elevated to 240 ng/ml of blasticidin ($P < 0.001$, *t*-test).
