# Supplementary material for: Puf Mediates Translation Repression of Transmission-Blocking Vaccine Candidates in Malaria Parasites
Source: PLoS Pathog. 2013 Apr 18;9(4):e1003268. doi: 10.1371/journal.ppat.1003268 (PMC3630172; doi:10.1371/journal.ppat.1003268)
Supplement: Figure S3 — Recombinant PfPuf2 binds to pfs25 5′UTR and pfs28 3′UTR in the presence of poly(U) as a competitor. ESMA of six RNA oligos from the pfs25 5′UTR and five RNA oligos from the pfs28 3′UTR (Figure S1) using the same conditions as in Figure 7 except that 100 nM of poly(U) was included in each reaction as a non-specific competitor. The Drosophila NRE sequence was used as a positive control. Lower bands in each gel are the unbound RNA, whereas the upper bands are the protein-RNA complex. (PDF) [file ppat.1003268.s003.pdf]

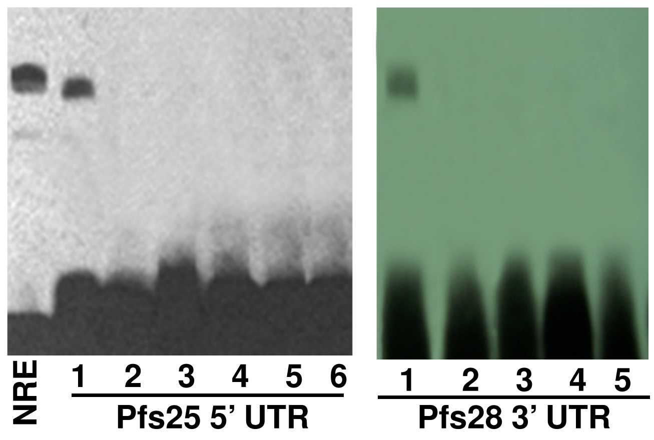

Figure S3. Recombinant PfPuf2 binds to *pfs25* 5'UTR and *pfs28* 3'UTR in the presence of poly(U) as a competitor. ESMA of six RNA oligos from the *pfs25* 5'UTR and five RNA oligos from the *pfs28* 3'UTR (Fig. S1) using the same conditions as in Fig. 7 except that 100 nM of poly(U) was included in each reaction as a non-specific competitor. The *Drosophila NRE* sequence was used as a positive control. Lower bands in each gel are the unbound RNA, whereas the upper bands are the protein-RNA complex.
