# Supplementary material for: Puf Mediates Translation Repression of Transmission-Blocking Vaccine Candidates in Malaria Parasites
Source: PLoS Pathog. 2013 Apr 18;9(4):e1003268. doi: 10.1371/journal.ppat.1003268 (PMC3630172; doi:10.1371/journal.ppat.1003268)
Supplement: Table S1 — Results from microarray analysis. (A) Up- and down-regulated transcripts in stage III gametocytes between 3D7 and ΔPfPuf2 line. (B) Up- and down-regulated transcripts in stage V gametocyte between 3D7 and ΔPfPuf2 line. (C) Up- and -down-regulated transcripts in both stage III and stage V gametocyte between 3D7 wild type and ΔPfPuf2 line. (D) Shared transcripts between up-regulated (>2 fold) in ΔPfPuf2 gametocytes (Stage III and V) and down-regulated in both ΔDOZ1 and ΔCITH in P. berghei gametocytes. (PDF) [file ppat.1003268.s004.pdf]

Table S1 A. Up- and down-regulated transcripts in stage III gametocytes between 3D7 and ΔPfPuf2 line.
*Gene IDs marked with * and annotation columns filled with red color are TR genes
t score differing for more than 2 standard deviations from the mean t-statistic value or fold change larger than 2 between average transcripts of 3d7 and ΔPfPuf2 are colored by dark blue, while 1.5 fold change by light blue

Enriched in 3D7 (235 genes)

Gende ID	Annotation	Functional Category	t-score#	Fold change in expression
PFC0400w	60S Acidic ribosomal protein P2	ribosomal	565.891	0.611
MAL13P1.262	hypothetical protein, conserved	apicoplast	541.038	0.439
PF11_0313	ribosomal phosphoprotein P0	ribosomal	516.147	0.593
PF13_0346	ubiquitin/ribosomal fusion protein uba52 homologue, putative	ribosomal	397.126	0.460
PF11_0043	60S acidic ribosomal protein p1, putative	ribosomal	357.038	0.706
PF14_0448	ribosomal protein S2, putative	ribosomal	337.489	0.498
PFB0885w	40S ribosomal protein S30, putative	ribosomal	288.313	0.600
PF08_0063	ClpB protein, putative	apicoplast, peptidase activity	288.279	0.276
PF14_0627	ribosomal protein S3, putative	ribosomal	279.551	0.420
PF10_0366	ADP/ATP transporter on adenylate translocase	mitochondrial	266.689	0.634
MAL7P1.100	serine/threonine protein kinase, Pfnek-4	Pr kinase	263.560	0.506
PF10_0144	prohibitin, putative	mitochondrial	250.821	0.542
PF14_0543	signal peptide peptidase, mSPP	apicoplast, peptidase	245.008	0.651
MAL13P1.480	histidine-rich protein III		225.355	0.046
PF13_0242	isocitrate dehydrogenase (NADP), mitochondrial precursor	mitochondrial	224.262	0.558
PF08_0076	40S ribosomal protein S16, putative	ribosomal	224.179	0.359
PFI1625c	organelle processing peptidase, putative	mitochondrial	222.403	0.421
PF14_0083	ribosomal protein S8e, putative	ribosomal	209.803	0.481
PFC0300c	60S ribosomal protein L7, putative	ribosomal	208.074	0.374
PFC0290w	40S ribosomal protein S23, putative	ribosomal	207.806	0.595
MAL13P1.164	elongation factor Tu, putative	mitochondrial	200.544	0.638
PFC0945w	protein kinase, putative	Pr kinase	198.682	0.318
PFL1015w	conserved Plasmodium protein, unknown function		198.678	0.503
PF11_0260	ribosomal protein L35, putative	ribosomal	198.529	0.519
PF13_0298	conserved Plasmodium protein, unknown function		198.422	0.638
PFE0890c	conserved Plasmodium protein, unknown function		192.041	0.652
PF10_0195a	flagellar outer arm dynein-associated protein, putative		189.375	0.643
MAL13P1.237	conserved Plasmodium protein, unknown function		188.363	0.587

PFB0455w	ribosomal L37ae protein, putative	ribosomal	188.349	0.474
PFC0381c	conserved Plasmodium protein, unknown function		186.474	0.540
PF13_0358	mitochondrial import inner membrane translocase, putative	mitochondrial	185.173	0.397
PF07_0074	conserved Plasmodium protein, unknown function		184.737	0.421
PFC0535w	60S ribosomal protein L26, putative	ribosomal	184.051	0.504
MAL13P1.270	proteasome subunit, putative	proteasome	181.838	0.520
PFB0295w	adenylosuccinate lyase, putative	DNA metabolism	181.667	0.429
MAL13P1.209	60S ribosomal subunit porotein L18, putative	ribosomal	179.593	0.655
PFE0495w	hypothetical protein, conserved	apicoplast	179.437	0.503
PF14_0231	ribosomal protein L7a, putative	ribosomal	178.213	0.470
PF13_0032	hydrolase, putative	apicoplast	174.322	0.435
PFI1216w	telomeric repeat binding factor 1		174.257	0.615
PF10_0203	ADP-ribosylation factor		171.452	0.572
PFL0210c	eukaryotic initiation factor 5a, putative		171.402	0.474
PFE0810c	40S ribosomal subunit protein S14, putative	ribosomal	170.750	0.419
PF14_0191	conserved Plasmodium protein, unknown function		169.562	0.469
PF13_0070	branched-chain alpha keto-acid dehydrogenase, putative	mitochondrial	168.962	0.399
PF14_0373	iron-sulphur protein subunit of the cytochrome bc1 complex	mitochondrial	168.015	0.467
PFA0225w	4-hydroxy-3-methylbut-2-enyl diphosphate reductase	apicoplast	165.564	0.442
PF13_0165	conserved Plasmodium protein, unknown function		164.357	0.739
PFC0975c	PFCYP19, cyclophilin, peptidyl-prolyl cis-trans isomerase		161.971	0.496
PF10_0245	glucosamine--fructose-6-phosphate aminotransferase, putative	apicoplast	159.773	0.368
PF11_0062	histone H2B, putative		158.012	0.618
PF13_0214	elongation factor 1-gamma, putative		156.676	0.602
PF08_0126	DNA repair protein rad54, putative	DNA metabolism	154.845	0.545
PFI0145w	hypothetical protein		153.284	0.540
PFD1055w	40S ribosomal protein S19, putative	ribosomal	153.233	0.396
MAL13P1.309	14-3-3 protein, putative		151.173	0.602
PF14_0597	cytochrome c1 precursor, putative	mitochondrial	150.525	0.400
PF14_0709	ribosomal protein L20, putative	ribosomal	147.541	0.463
PFB0795w	ATP synthase F1, alpha subunit, putative	mitochondrial	147.171	0.469
PF14_0471	transcription factor with AP2 domain(s), putative		146.915	0.645
PFE1595c	Plasmodium exported protein (PHISTc), unknown function		145.678	0.279
PF08_0075	60S ribosomal protein L13, putative	ribosomal	145.340	0.685
PF10_0063	DNA/RNA-binding protein Alba, putative		145.254	0.427
PF14_0240	ribosomal protein L21e, putative	ribosomal	144.616	0.500
PF14_0450	conserved Plasmodium protein, unknown function		137.116	0.670

PFE0285c	ubiquitin-like protein, putative		136.578	0.566
PF10_0218	citrate synthase, mitochondrial precursor, putative	mitochondrial	136.352	0.516
PF11_0282	deoxyuridine 5'-triphosphate nucleotidohydrolase, putative	DNA metabolism	135.638	0.431
PF13_0246	hypothetical protein		135.262	0.579
PFD1090c	clathrin assembly protein, putative		134.719	0.566
PFF0860c	histone H2A		134.604	0.675
PF11_0351	heat shock protein hsp70 homologue	mitochondrial	133.320	0.477
PFL1555w	cytochrome b5, putative		131.256	0.656
PFI1735c	ring-exported protein 1		129.721	0.098
PF10_0067	hypothetical protein		129.184	0.714
PF14_0296	60S ribosomal protein L14, putative	ribosomal, apicoplast	128.753	0.391
PF13_0011	plasmodium falciparum gamete antigen 27/25		128.665	0.649
PFC0371w	hypothetical protein		128.135	0.474
PFB0790c	hypothetical protein		126.678	0.623
PF13_0129	ribosomal protein L6 homologue, putative	ribosomal	126.605	0.611
PFI1365w	cytochrome c oxidase subunit, putative	mitochondrial	126.472	0.521
PFD0470c	replication factor a protein, putative	DNA replicaton	126.265	0.506
PFA0290w	DNA binding protein, putative		126.016	0.627
PF14_0784	ribosome biogenesis protein, NOP10-like	ribosomal	125.357	0.552
PF13_0268	ribosomal protein L17, putative	ribosomal	125.106	0.389
MAL8P1.122	ubiquitin regulatory protein, putative		124.975	0.761
PF11_0214	conserved Plasmodium protein, unknown function		124.805	0.366
PF07_0096	conserved Plasmodium protein, unknown function		124.754	0.435
MAL7P1.300	40S ribosomal protein S29, putative	ribosomal	124.642	0.751
PFB0115w	conserved Plasmodium protein, unknown function		123.929	0.106
MAL7P1.24	large ribosomal subunit processing protein, putative	ribosomal	122.598	0.575
PF11_0198	tRNA m(1)G methyltransferase, putative		121.528	0.682
PFB0880w	hypothetical protein, conserved		121.482	0.628
PF08_0122	conserved Plasmodium protein, unknown function		120.775	0.685
PFE0480c	hypothetical protein, conserved	apicoplast	120.209	0.604
PF14_0592	conserved Plasmodium protein, unknown function		119.028	0.518
PFL1095c	conserved Plasmodium protein, unknown function		117.837	0.744
PFL2290w	preprocathepsin c precursor, putative		117.756	0.745
PFC0710w.1	inorganic pyrophosphatase, putative(-a)?		117.434	0.693
PFD1100c	hypothetical protein, conserved	apicoplast	116.826	0.708
PF07_0079	60S ribosomal protein L11a, putative	ribosomal	115.857	0.585
PF13_0139	hypothetical protein, conserved		114.428	0.412

PFC0795w	hypothetical protein, conserved	apicoplast	113.687	0.395
PFD0785c	hypothetical protein		113.398	0.710
PF11_0442	transcription factor with AP2 domain(s), putative		113.330	0.739
MAL13P1.332	hypothetical protein, conserved	apicoplast	112.793	0.679
PFL1840w	conserved Plasmodium membrane protein, unknown function		111.513	0.587
PF07_0091	cell cycle control protein cwf15 homologue		111.077	0.756
PFL2285c	conserved Plasmodium protein, unknown function	tetrapyrrole biosynthetic process	111.073	0.557
PFI1075w	conserved Plasmodium protein, unknown function		110.479	0.558
PFF0760w	RNA and export factor binding protein, putative		109.833	0.580
PFD0580c	conserved Plasmodium protein, unknown function		109.115	0.630
PFL2405c	PFG377 protein, osmiophilic body protein	apicoplast	108.364	0.212
PFA0475c	conserved Plasmodium protein, unknown function		108.268	0.575
PF14_0509	RAP protein, putative		106.848	0.291
PF14_0586a	conserved Plasmodium protein, unknown function		106.792	0.480
PFL0800c	cell traversal protein for ookinetes and sporozoites		106.304	0.445
MAL13P1.111	ATP-dependent Clp protease adaptor protein ClpS, putative		105.867	0.414
PFD0993c	conserved Plasmodium protein, unknown function	protein amino acid dephosphorylation	105.469	0.721
MAL8P1.77	conserved Plasmodium protein, unknown function		105.156	0.182
MAL13P1.233	DNA/RNA-binding protein Alba, putative		104.715	0.512
PFF0640w	conserved Plasmodium protein, unknown function		104.361	0.542
PFL0510c	conserved Plasmodium protein, unknown function		103.694	0.484
PF13_0058	hypothetical protein		103.395	0.782
PF14_0585	ribosomal protein S28e, putative	ribosomal	103.279	0.776
PF14_0805	conserved Plasmodium protein, unknown function		102.834	0.777
PFB0601c	conserved Plasmodium protein, unknown function		102.639	0.667
PF14_0248	ubiquinol-cytochrome c reductase hinge protein, putative	mitochondrial	102.523	0.686
PF14_0524	protein phosphatase, putative		102.397	0.612
PFI1415w	Serine/Threonine protein kinase, putative	Pr kinase	102.213	0.638
MAL13P1.350	conserved Plasmodium protein, unknown function		102.046	0.547
PFC0380w	dual-specificity protein phosphatase, putative		101.934	0.594
PF10_0062	NOT family protein, putative		101.454	0.547
MAL7P1.320	ribosomal protein, L37e, putative	ribosomal	101.443	0.714
PFE0845c	60S ribosomal subunit protein L8, putative	ribosomal	101.299	0.588
PFA0340w	2-C-methyl-D-erythritol 4-phosphate cytidylyltransferase, putative	apicoplast	101.101	0.586
PFE1173c	outer arm dynein lc3, putative		100.459	0.607
PFC0710w.2	inorganic pyrophosphatase, putative(-a)?		100.336	0.714
PF08_0097	conserved Plasmodium membrane protein, unknown function	transport	100.207	0.450

PF07_0054	histone H2B variant, putative		99.657	0.718
PFF0725w	conserved Plasmodium protein, unknown function		99.393	0.600
PFE0770w	conserved Plasmodium protein, unknown function		99.075	0.544
PF08_0039	60S ribosomal protein L22, putative	ribosomal	98.753	0.621
PFL1290w	hypothetical protein		98.213	0.508
PF14_0018	Plasmodium exported protein (PHISTb), unknown function		97.833	0.764
PFI1325w	CS domain protein, putative		97.532	0.563
PF11_0183	GTP-binding nuclear protein ran/tc4	DNA metabolism	96.250	0.729
PFE0310c	conserved Plasmodium protein, unknown function	regulation of transcription, DNA-dependent	96.218	0.461
PFA0360c	hypothetical protein conserved in P. falciparum		95.854	0.620
PFD0485w	conserved Plasmodium protein, unknown function	regulation of transcription, DNA-dependent	95.103	0.486
PF10_0066	thioredoxin, putative		95.083	0.680
PF10_0043	60S ribosomal protein L13, putative	ribosomal	94.796	0.505
PF10_0095	conserved Plasmodium membrane protein, unknown function		93.996	0.734
PF14_0575a	conserved Plasmodium membrane protein, unknown function		93.841	0.333
PF13_0082	cop-coated vesicle membrane protein p24 precursor, putative		93.595	0.503
PFA0425c	conserved Plasmodium membrane protein, unknown function		93.169	0.729
MAL8P1.70	conserved Plasmodium protein, unknown function		92.899	0.454
PFF0395c	conserved Plasmodium membrane protein, unknown function		92.693	0.561
PFC0315c	conserved Plasmodium protein, unknown function		92.618	0.583
PF14_0617	conserved hypothetical protein		92.278	0.752
PFI1618w	conserved Plasmodium protein, unknown function		92.107	0.523
PF11_0054	sun-family protein, putative		91.965	0.602
PFL1475w	conserved Plasmodium protein, unknown function		91.223	0.636
PFF1170w	conserved hypothetical protein		91.206	0.617
PF14_0291	telomerase reverse transcriptase, putative		89.837	0.639
PF13_0080	conserved hypothetical protein		89.403	0.499
PF14_0705	hypothetical protein	apicoplast	89.083	0.435
PF11_0372	conserved Plasmodium protein, unknown function	transcription initiation from RNA polymerase II promote	89.016	0.614
PF11_0458	hypothetical protein, conserved	apicoplast	88.825	0.669
MAL13P1.49	secretory complex protein 61 beta subunit		88.660	0.282
MAL8P1.51	DEAD-box RNA helicase, putative		87.610	0.314
PF14_0563	sedlin, putative		87.352	0.790
PF13_0174	step II splicing factor, putative		87.290	0.530
PFF0500c	conserved Plasmodium protein, unknown function		87.164	0.523
PFL0225c	circumsporozoite-related antigen		87.117	0.402
PF11_0224	RNA-binding protein s1, putative		86.889	0.705

PF11_0320	vacuolar ATP synthase subunit D, putative		86.225	0.660
PF13_0227	cold-shock protein, putative	DNA metabolism	86.144	0.369
PFA0470c	conserved Plasmodium protein, unknown function		85.956	0.481
MAL8P1.10	RNA polymerase subunit 8c, putative		85.618	0.602
PFL0665c	hypothetical protein	apicoplast	85.548	0.395
PF11_0323	iron-sulfur subunit of succinate dehydrogenase	mitochondrial	85.302	0.655
PFL0630w	conserved Plasmodium protein, unknown function		85.287	0.894
PF14_0336	glycine --tRNA ligase, putative	apicoplast, DNA metabolism	85.251	0.563
PF14_0198	conserved Plasmodium protein, unknown function		85.230	0.550
PF13_0342	conserved Plasmodium protein, unknown function		84.223	0.645
PF11_0199	conserved Plasmodium protein, unknown function		83.883	0.794
PF10_0076	Thiamine pyrophosphokinase	apicoplast	83.520	0.670
PFI1195c	conserved Plasmodium protein, unknown function		82.725	0.605
PF14_0815	conserved Plasmodium protein, unknown function		82.456	0.645
PF14_0092	conserved Plasmodium protein, unknown function		81.912	0.745
PFL0980w	ubiquitin-conjugating enzyme, putative		81.893	0.742
PF10_0330	conserved Plasmodium protein, unknown function		81.822	0.817
MAL7P1.29	conserved Plasmodium protein, unknown function	translation / translational initiation	80.531	0.773
PF10_0279	conserved Plasmodium protein, unknown function	regulation of transcription, DNA-dependent	80.294	0.836
PFE0275w	mitochondrial ribosomal protein L19 precursor, putative	ribosomal	80.052	0.423
PFF0495w	microtubule-associated protein 1 light chain 3, putative		79.969	0.656
PF10_0193	ubiquitin--protein ligase, putative		79.848	0.456
PFC0845c	cyclophilin, putative		79.795	0.779
PF11_0170	hypothetical protein, conserved	apicoplast	79.710	0.687
PFC0925w	merozoite surface protein 3		79.377	0.634
PF10_0345	hypothetical protein	apicoplast	79.312	0.158
PF14_0367	hypothetical protein		79.204	0.555
PF10_0283	ubiquitin conjugating enzyme, putative		79.166	0.498
PFI0740c	RAP protein, putative		78.262	0.712
PFE0800w	conserved Plasmodium protein, unknown function	regulation of transcription, DNA-dependent	78.230	0.490
PFL2340w	serine/threonine protein kinase, FIKK family	Pr kinase	78.127	0.539
PFI0110c	exosome rRNA processing protein, putative	DNA metabolism	78.044	0.225
PFD0515w	SFT2-like protein, putative		77.686	0.434
PF13_0124	hypothetical protein		77.168	0.686
PFB0391c	conserved Plasmodium protein, unknown function		76.962	0.665
PF11_0196	nonclathrin coat protein zeta2-cop-related protein, putative		76.940	0.708
PFD0745c	conserved Plasmodium protein, unknown function		76.910	0.900

PFB0425c	conserved Plasmodium protein, unknown function		76.821	0.484
PFE0155w	exonuclease i, putative	DNA metabolism	76.693	0.749
PF07_0105	hypothetical protein, conserved		76.684	0.644
PF11_0474	signal recognition particle SRP14		76.003	0.739
PFL0160w	Antigen UB05		75.831	0.444
PF10_0372	conserved Plasmodium protein, unknown function		75.624	0.873
PF14_0644	hypothetical protein		75.297	0.563
PFB0590w	conserved Plasmodium protein, unknown function		74.995	0.757
PFL2145w	deoxyhypusine hydroxylase	mitochondrial	74.798	0.606
PF13_0013	high mobility group protein		74.748	0.711
PFL0145c	conserved Plasmodium protein, unknown function		74.698	0.732
PFD0520c	conserved Plasmodium protein, unknown function		74.638	0.632
PF13_0020	hypothetical protein	apicoplast	74.603	0.620
PF14_0435	Translation initiation factor SUI1, putative		74.485	0.587
PFL2095w	hypothetical protein, conserved		74.395	0.811
MAL8P1.25	long chain polyunsaturated fatty acid elongation enzyme, putative		74.391	0.360
PFF0290w	receptor for activated c kinase	Pr kinase	74.082	0.663
PF08_0019	hypothetical protein		74.047	0.746
PF11_0296	small GTP-binding protein sar1		73.888	0.559
PFD0810w	conserved Plasmodium protein, unknown function		73.301	0.775
MAL13P1.215	Plasmodium exported protein (hyp10), unknown function	apicoplast	73.092	0.575
PFA0700c	conserved Plasmodium protein, unknown function		73.054	0.206

Enriched in ΔPuf2 (226 genes)

Gende ID	Annotation	Functional Category	t-score	Fold change in expression
PFE0315c	conserved Plasmodium protein, unknown function		-67.970	1.754
PFC0595c	conserved Plasmodium protein, unknown function		-67.977	1.641
PF11_0479	serine/threonine protein phosphatase, putative		-68.106	1.559
PFL1895w	conserved Plasmodium protein, unknown function		-68.172	1.850
PF13_0247	mitochondrial ribosomal protein L23 precursor, putative	ribosomal	-68.191	2.472
PF14_0602	6-cysteine protein		-68.204	2.317
PF07_0088	DNA polymerase alpha subunit, putative	DNA polymerase, DNA replicaton	-68.290	1.861
MAL13P1.220	40S ribosomal protein S5, putative	ribosomal	-68.324	1.693
PFF1410c	lipoate synthase, putative	apicoplast	-68.441	1.328
PFC0475c	nicotinate phosphoribosyltransferase, putative		-68.566	1.505

PF11_0508	phosphatidylinositol 3- and 4-kinase, putative			-68.579	1.468
PF08_0102	Plasmodium exported protein, unknown function			-68.636	2.496
PF07_0109	asparagine-rich antigen Pfa55-14			-68.664	2.392
PFA0285c	hypothetical protein, conserved		apicoplast	-68.802	1.338
PF08_0078	conserved Plasmodium protein, unknown function	cell cycle		-68.808	2.912
PFE0875c	ABC transporter, putative		apicoplast	-68.868	1.501
PFC0745c	hypothetical protein, conserved		apicoplast	-69.005	1.603
PFL1785c	proteasome component C8, putative		proteasome	-69.026	1.278
PF08_0054	conserved Plasmodium protein, unknown function	cell cycle		-69.054	1.272
PF08_0129	heat shock 70 kDa protein			-69.240	2.309
PF14_0667	serine/threonine protein phosphatase, putative			-69.330	1.411
PF11_0177	hypothetical protein			-69.397	1.375
PF10_0268	deubiquinating/deneddylating enzyme		peptidase activity	-69.408	1.275
PFA0295c	merozoite capping protein 1			-69.517	1.840
PF08_0086	conserved Plasmodium protein, unknown function			-69.557	1.411
PF10_0244	RNA binding protein, putative			-69.563	1.377
PF14_0021	formin 2, putative			-69.567	1.356
PF14_0035	conserved Plasmodium protein, unknown function			-70.025	2.078
PFL2065c	nuclear formin-like protein			-70.361	2.070
PF14_0571	mitochondrial import inner membrane translocase subunit, putative		mitochondrial	-70.789	1.325
PF08_0096	conserved Plasmodium membrane protein, unknown function			-71.039	2.404
PF14_0442	RNA helicase, putative			-71.052	2.264
PFD0400w	conserved Plasmodium membrane protein, unknown function			-71.249	1.943
PFC0895w	conserved Plasmodium protein, unknown function			-71.406	2.320
PFB0490c	CPW-WPC family protein			-71.635	1.300
PFC0570c	conserved hypothetical protein			-71.848	1.243
PFF0540c	conserved hypothetical protein			-71.887	1.851
PFE0220w	conserved Plasmodium membrane protein, unknown function			-71.901	1.844
PFI1095w	conserved Plasmodium protein, unknown function	attachment of GPI anchor to protein		-72.317	1.269
MAL13P1.287	conserved Plasmodium protein, unknown function	attachment of GPI anchor to protein		-72.354	1.683
MAL13P1.224	conserved Plasmodium protein, unknown function	attachment of GPI anchor to protein		-72.417	1.355
MAL13P1.55	conserved Plasmodium protein, unknown function	attachment of GPI anchor to protein		-72.480	2.621
PFL0150w	cytochrome c2 precursor,putative		mitochondrial	-72.554	1.911
PFL1400c	origin recognition complex 1 protein		DNA replicaton	-73.035	1.812
PFL1865w	conserved Plasmodium protein, unknown function			-73.039	2.078
PFB0190c	conserved Plasmodium protein, unknown function			-73.055	1.739
PFD0885c	conserved Plasmodium protein, unknown function			-73.337	1.674

PF11_0374	conserved Plasmodium protein, unknown function		-73.541	1.302
PF10_0061	tudor staphylococcal nuclease		-73.825	1.202
PFL1170w	conserved Plasmodium protein, unknown function		-74.312	2.203
PFA0535c	polyadenylate-binding protein, putative		-74.327	1.725
MAL7P1.25	kinesin, putative	dynein	-74.512	1.665
PF14_0178	cytoskeleton associated protein, putative		-74.684	2.205
PFL0305c	ubiquitin fusion degradation protein UFD1, putative		-74.800	1.453
PF13_0315	IMP-specific 5'-nucleotidase, putative,haloacid dehalogenase hydrolase, pu	DNA metabolism	-75.232	1.258
PF13_0072	rRNA associated RNA binding protein, putative		-75.887	1.656
MAL13P1.278	conserved Plasmodium protein, unknown function		-76.513	1.993
PF14_0338	Ser/Thr protein kinase	Pr kinase	-76.686	2.325
PFL0120c	conserved hypothetical protein		-77.023	1.498
MAL13P1.229	cyclophilin, putative		-77.097	1.654
PFD0170c	conserved hypothetical protein		-77.205	1.368
PFL0130c	conserved hypothetical protein		-77.543	1.950
PF14_0088	hypothetical protein, conserved	mitochondrial	-77.584	2.260
PFF1200w	aldo-keto reductase, putative	apicoplast	-77.626	1.352
PF14_0127	conserved Plasmodium protein, unknown function		-78.060	1.520
MAL7P1.91	N-myristoyltransferase		-79.108	1.948
PFF0720w	exported serine/threonine protein kinase	Pr kinase	-79.562	1.364
PF10_0155	conserved Plasmodium membrane protein, unknown function		-79.777	2.080
PF14_0123	enolase		-79.855	1.870
PF13_0104	hypothetical protein		-79.966	1.272
PF14_0315	hypothetical protein		-80.038	1.410
PFL1125w	conserved Plasmodium membrane protein, unknown function		-80.456	2.151
PFE0395c	phospholipid-transporting ATPase, putative		-81.234	1.248
MAL13P1.145	6-cysteine protein		-81.791	1.567
PFL1890c	conserved Plasmodium protein, unknown function		-81.990	1.814
PF14_0024	HD superfamily phosphohydrolase protein		-82.628	1.218
PFC0705c	hypothetical protein		-82.689	1.334
PF14_0540	conserved Plasmodium protein, unknown function	microtubule cytoskeleton organization	-84.238	1.565
PF14_0624	conserved Plasmodium protein, unknown function	protein import into nucleus	-84.281	1.682
PF13_0188	conserved Plasmodium protein, unknown function	protein import into nucleus	-84.438	1.645
PF14_0281	conserved Plasmodium protein, unknown function		-84.786	1.661
PFB0815w	plasmepsin IX	peptidase activity	-85.315	2.198
PF14_0696	calcium dependent protein kinase 1	Pr kinase	-85.452	1.521
PF14_0227	hypothetical protein	apicoplast	-85.484	1.654

PFD1115c	calcium-dependent protein kinase, putative	Pr kinase	-88.108	1.692
PF11_0377	conserved Plasmodium protein, unknown function		-88.733	1.408
PF11_0307	casein kinase 1, PfCK1	Pr kinase	-89.343	1.268
PF11_0292	phosphatidylinositol-4-phosphate-5-kinase, putative	apicoplast	-89.619	1.375
PFD0700c	cochaperone prefoldin complex subunit, putative		-89.679	2.420
PF10_0164	RNA binding protein, putative	DNA metabolism	-89.885	1.266
PF13_0048	early transcribed membrane protein 10.3, etramp10.3		-90.282	1.887
PFL0440c	NUDIX hydrolase, putative		-90.320	1.483
PF14_0020	zinc finger protein, putative		-90.608	1.482
PF13_0241	choline kinase, putative	kinase	-90.623	1.407
PF13_0216	rhomboid protease ROM6, putative	mitochondrial	-91.026	1.543
PF10_0210	conserved Plasmodium protein, unknown function		-91.556	1.508
PFC0435w	deoxyribose-phosphate aldolase, putative	DNA metabolism	-91.612	1.830
PF14_0486	parasite-infected erythrocyte surface protein	apicoplast	-91.909	1.648
PF11_0266a	elongation factor 2		-92.002	1.780
PF14_0446	conserved Plasmodium protein, unknown function		-92.173	1.340
PF14_0544	heme detoxification protein		-92.703	2.117
PFI0940c	conserved Plasmodium protein, unknown function		-93.166	2.100
PFB0685c	PPPDE peptidase, putative	peptidase	-93.598	1.295
PF10_0165	acyl-CoA synthetase, PfACS9	apicoplast	-93.774	2.318
PF07_0035	DNA polymerase delta catalytic subunit	DNA polymerase, DNA replicaton	-94.595	1.583
PF14_0249	Cg1 protein		-94.818	1.425
PFC0915w	hypothetical protein	apicoplast	-95.068	1.357
PF11_0362	ATP-dependent RNA helicase, putative		-95.137	1.762
PF14_0056	protein phosphatase, putative		-95.162	1.269
PFE0830c	RNA binding protein, putative		-96.309	2.679
MAL13P1.328	sporozoite surface antigen MB2	apicoplast	-96.367	1.672
PF13_0201	DNA topoisomerase VI, b subunit, putative	DNA replicaton, DNA metabolism	-96.400	1.889
PF13_0226*	conserved Plasmodium protein, unknown function		-96.747	1.452
MAL7P1.102	Pfs77 protein-related		-96.906	1.847
PFL1235c	sporozoite surface protein 2 or Thrombospondin-related anonymous protein, TRAP		-97.037	1.322
PF14_0427	conserved Plasmodium protein, unknown function		-97.055	1.275
PFE0930w	conserved Plasmodium protein, unknown function		-97.124	1.396
PF14_0523	WD-repeat protein, putative		-97.372	1.573
PF10_0295	protein phosphatase 2C, putative		-97.570	1.275
PF14_0375	hypothetical protein		-97.872	1.670
PFL0295c	hypothetical protein		-98.185	1.695

PFA0255c	hypothetical protein, conserved		-98.387	2.182
PF08_0080	conserved Plasmodium protein, unknown function		-98.426	1.831
PFF0450c	conserved Plasmodium protein, unknown function		-98.981	1.555
PFL0445w	Zn2+ or Fe2+ permease		-99.198	2.394
PFL0110c	conserved Plasmodium protein, unknown function		-99.435	1.598
PF13_0328	mitochondrial phosphate carrier protein, PfmpC	mitochondrion	-99.545	1.598
PFD0770c	proliferating cell nuclear antigen	DNA replicaton, DNA metabolism	-102.094	1.323
PFL0700w	60S ribosomal protein L15, putative	ribosomal	-102.726	1.824
PFB0895c	conserved hypothetical protein	apicoplast	-103.191	2.549
PF13_0035	replication factor C subunit 1, putative	DNA replicaton, DNA metabolism	-103.451	1.408
PFL2060c	U3 small nucleolar RNA-associated protein 6, putative		-103.477	2.133
MAL8P1.209	rab specific GDP dissociation inhibitor		-103.912	1.967
PFL1610c	var-like protein		-103.992	2.007
PF11_0208	hypothetical protein, conserved		-104.226	1.949
PFE1150w	phosphoglycerate mutase, putative		-104.389	2.298
MAL13P1.313	multidrug resistance protein		-104.614	1.879
PF11_0084	conserved Plasmodium protein, unknown function		-105.666	2.073
PF11_0342	conserved Plasmodium protein, unknown function		-105.855	1.438
PFE0530w	conserved Plasmodium protein, unknown function		-108.254	1.771
PF11_0049	conserved Plasmodium protein, unknown function		-108.881	2.687
PF14_0162	NOT family protein, putative		-109.689	1.524
MAL13P1.195*	CPW-WPC family protein		-111.774	1.590
PFF0305c	conserved Plasmodium protein, unknown function		-112.153	1.660
PF10_0179a	ubiquitin conjugating enzyme E2, putative		-113.889	1.663
PFD1050w	conserved Plasmodium protein, unknown function		-114.032	2.679
PFI0185w	alpha tubulin 2		-115.543	1.514
PF07_0077	LCCL domain-containing protein	apicoplast	-115.788	2.086
PF11_0097	actin-like protein, putative		-115.974	1.531
PF10_0332	succinyl-CoA synthetase alpha subunit, putative	mitochondrial	-116.182	5.042
PF14_0633a	apicoplast ribosomal protein L27 precursor, putative	ribosomal, apicoplast	-117.124	1.800
PF14_0492	conserved Plasmodium protein, unknown function		-117.362	1.933
PFE0715w	protein phosphatase 2b regulatory subunit, putative		-119.469	1.690
PF14_0437	asparagine --tRNA ligase, putative	DNA metabolism, apicoplast	-119.602	2.090
PFF1340w	DEAD/DEAH box ATP-dependent RNA helicase, putative		-119.938	1.987
PF14_0690	conserved Plasmodium protein, unknown function		-120.427	2.082
PF13_0349	histone deactylase, putative	DNA metabolism	-120.485	1.832
PFF1180w	nucleoside diphosphate kinase b; putative	DNA metabolism, kinase	-120.901	2.080

PF11_0253	anaphase-promoting complex subunit, putative		-120.915	2.157
PF14_0538	conserved Plasmodium protein, unknown function		-122.524	1.717
PFI0440w	conserved Plasmodium protein, unknown function		-123.827	1.481
PF13_0161	conserved Plasmodium protein, unknown function		-124.196	2.008
PFB0525w	conserved Plasmodium protein, unknown function		-124.392	1.285
PF13_0168	asparagine -- tRNA ligase, putative	DNA metabolism	-125.619	1.966
MAL7P1.117	CPW-WPC family protein	apicoplast	-126.112	1.509
PF11_0535	conserved Plasmodium protein, unknown function		-126.968	1.611
PFL1295w	conserved Plasmodium protein, unknown function		-128.318	1.424
PFD0795w	hypothetical protein, conserved		-128.671	1.514
PF10_0194	histone acetyltransferase, putative		-128.840	1.944
PF11_0464	large subunit rRNA processing RRM protein, putative		-129.901	1.552
PFC0495w*	plasmepsin VI	peptidase activity	-130.039	1.751
PFD0820w	serine/threonine protein kinase	apicoplast	-130.162	1.448
MAL13P1.42	conserved Plasmodium protein, unknown function	apicoplast	-130.295	2.455
PF11_0331	recombinase, putative	DNA replicaton	-130.337	1.763
PFL1990c	TCP-1/cpn60 chaperonin family, putative		-131.218	2.004
PF11_0193	conserved Plasmodium protein, unknown function		-132.047	1.780
MAL13P1.420	conserved Plasmodium protein, unknown function		-133.307	3.246
MAL8P1.17	protein disulfide isomerase		-133.485	1.396
PFF1035w	Pf77 protein		-137.366	1.332
PF14_0323	calmodulin	dynein	-138.334	1.549
PF14_0598	glyceraldehyde-3-phosphate dehydrogenase		-139.194	2.311
MAL13P1.130	glideosome associated protein with multiple membrane spans 1		-142.449	2.321
MAL7P1.156	conserved Plasmodium protein, unknown function		-142.511	1.558
PFF0220w	conserved Plasmodium protein, unknown function		-143.174	2.501
PF07_0023	minchromosome maintenance (MCM) complex subunit, putative	DNA replicaton	-144.176	1.996
PF11_0160	SET domain protein, putative		-146.583	1.389
PF10_0242	conserved Plasmodium protein, unknown function		-148.023	1.645
PFI1350c	dynein light chain, putative	dynein	-148.943	1.600
MAL13P1.96	chromosome segregation protein, putative	dynein, DNA replicaton	-152.526	1.873
PFB0405w	transmission-blocking target antigen s230 precursor		-156.377	2.534
PF10_0302*	ookinete surface protein Pos28-1, putative		-156.553	1.716
PFA0170c	zinc-carboxypeptidase, putative		-156.742	1.906
MAL7P1.17	conserved Plasmodium membrane protein, unknown function		-156.774	2.224
PF14_0425	fructose-bisphosphate aldolase		-157.579	1.921
PFC0860w	kinesin, putative	dynein	-158.290	1.728

PF14_0792	conserved Plasmodium protein, unknown function		-158.415	3.494
PF07_0072	calcium-dependent protein kinase 4	Pr kinase	-159.921	2.726
MAL13P1.168	protein tyrosine phosphatase, putative		-160.534	2.124
PFI1285w	protein kinase, putative	Pr kinase, apicoplast	-163.248	1.450
PF13_0144	oxidoreductase, putative		-171.377	2.606
PF14_0243a	dynein-associated protein, putative	dynein	-174.930	2.528
PFE0415w	transcription factor iib, putative	DNA metabolism	-179.296	1.959
PF11_0092	mechanosensitive ion channel protein		-180.869	2.457
PF07_0087	hypothetical protein, conserved	apicoplast	-184.698	2.907
PF14_0601	replication factor C3	DNA replicaton	-190.368	2.909
PFF0875w	conserved Plasmodium protein, unknown function		-194.406	1.237
PF13_0269	glycerol kinase, putative	kinase	-195.193	1.382
PF14_0359	HSP40, subfamily A, putative		-196.448	1.692
PFD0420c	flap exonuclease, putative	DNA replicaton	-198.953	3.669
PFE0870w	transcriptional regulator, putative	DNA metabolism	-200.872	1.960
PF13_0095	minchromosome maintenance (MCM) complex subunit, putative	DNA replicaton	-205.172	2.677
PFC0260w	P-loop containing nucleoside triphospahte hydrolase, putative	apicoplast	-212.287	2.439
PF14_0030	hypothetical protein	dynein, apicoplast	-217.427	1.406
PF11_0148	dynein light chain type 2, putative	dynein	-223.922	2.078
MAL7P1.74	secreted ookinete protein, putative		-230.159	2.466
PF14_0366	small subunit DNA primase, putative	DNA replicaton	-236.173	2.318
PFI0690c	conserved Plasmodium protein, unknown function		-236.887	2.435
PFE0675c	deoxyribodipyrimidine photolyase (photoreactivating enzyme, DNA photol	DNA metabolism	-249.171	1.904
PF07_0029	heat shock protein 90		-249.677	2.266
PF14_0712	hypothetical protein	apicoplast	-250.466	2.237
PF14_0504a	conserved Plasmodium protein, unknown function		-255.740	3.986
PF10_0303*	25 kDa ookinete surface antigen precursor (pfs25)		-260.851	8.147
PF11_0057	conserved Plasmodium protein, unknown function	dynein	-305.183	2.026
MAL7P1.125	conserved Plasmodium protein, unknown function		-327.592	2.061
PF14_0177	minchromosome maintenance (MCM) complex subunit	DNA replicaton	-328.354	2.671
PF14_0053	ribonucleotide reductase small subunit	DNA replicaton	-367.222	2.235

Table S1 B. Up- and down-regulated transcripts in stage V gametocyte between 3D7 and ΔPfPuf2 line.

*Gene IDs marked with * and annotation columns filled with red colour are TR genes

t score differing for more than 2 standard deviations from the mean t-statistic value or fold change larger than 2 between average transcripts of 3d7 and ΔPfPuf2 are colored by dark blue, while 1.5 fold change by light blue

Enriched in 3D7 (193 genes)

Gende ID	Annotation	Functional Category	t-score #	Fold change in expression
MAL13P1.480	histidine-rich protein III		731.429	0.010
MAL13P1.164	elongation factor tu, putative	mitochondrial	551.273	0.355
PFI1735c	ring-exported protein 1		548.061	0.031
PFC0400w	60S Acidic ribosomal protein P2, putative	ribosomal	534.206	0.544
PF11_0043	60S acidic ribosomal protein p1, putative	ribosomal	494.059	0.622
PF13_0346	hypothetical protein	ribosomal	435.628	0.405
PF08_0126	hypothetical protein	DNA metabolism	366.489	0.524
PF11_0097	hypothetical protein	mitochondrial	366.446	0.479
PFB0295w	adenylosuccinate lyase, putative	DNA metabolism	334.119	0.392
PFE0845c	hypothetical protein	ribosomal	326.919	0.427
MAL7P1.100	serine/threonine protein kinase 2, putative	Pr kinase	325.339	0.563
PF10_0210	deoxyribose-phosphate aldolase, putative	DNA metabolism	303.252	0.543
PF08_0054	heat shock 70 kDa protein, putative		292.104	0.712
PF07_0079	60S ribosomal protein L11a, putative	ribosomal	286.379	0.123
PF14_0230	Ribosomal protein family L5, putative	ribosomal	281.295	0.546
MAL7P1.231	histidine-rich protein II		276.320	0.106
PFE1595c	Plasmodium exported protein (PHISTc), unknown function		266.493	0.150
PF13_0032	conserved hypothetical protein	apicoplast	245.898	0.604
PF13_0268	ribosomal protein L17, putative	ribosomal	240.857	0.427
PFF0860c	histone H2A		235.078	0.722
PFB0455w	ribosomal L37ae protein, putative	ribosomal	234.657	0.556
PF10_0144	prohibitin, putative	mitochondrial	234.071	0.612
PF08_0076	40S ribosomal protein S16, putative	ribosomal	231.912	0.320
PFB0115w	conserved Plasmodium protein, unknown function		219.129	0.059
PF13_0242	isocitrate dehydrogenase (NADP), mitochondrial precursor,	mitochondrial	217.864	0.517

PFE0495w	conserved hypothetical protein	apicoplast	207.150	0.551
PF10_0366	ADP/ATP transporter on adenylate translocase, putative	mitochondrial	205.855	0.390
PFE0480c	hypothetical protein	apicoplast	193.153	0.710
PF14_0597	cytochrome c1 precursor, putative	mitochondrial	189.747	0.500
PF14_0333	hypothetical protein		189.696	0.811
PF07_0080	40S ribosomal protein S10, putative	ribosomal	188.502	0.423
PFL0385c	blood stage antigen 41-3 precursor		184.679	0.524
PF13_0011	plasmodium falciparum gamete antigen 27/25		177.426	0.635
PF10_0345	merozoite surface protein 3		177.381	0.175
PF13_0076	Plasmodium exported protein, unknown function		169.649	0.498
PFD1055w	ribosomal protein S19s, putative	ribosomal	162.478	0.616
PFI1625c	organelle processing peptidase, putative	peptidase, mitochondrial	160.941	0.446
PF11_0535	conserved Plasmodium protein, unknown function		160.554	0.692
PF11_0351	heat shock protein hsp70 homologue	mitochondrial	158.346	0.593
PF14_0240	ribosomal protein L21e, putative	ribosomal	157.960	0.470
PFE0060w	parasite-infected erythrocyte surface protein		154.981	0.038
PF13_0129	ribosomal protein L6 homologue, putative	ribosomal	154.435	0.627
PFL1675c	CPW-WPC family protein		154.140	0.372
PF14_0083	ribosomal protein S8e, putative	ribosomal	153.697	0.486
MAL8P1.16	rhomboid protease ROM3	peptidase	152.899	0.646
PF10_0026	tryptophan-rich antigen 3		147.778	0.041
PF08_0059	protein kinase c inhibitor-like protein, putative	kinase	147.122	0.530
PFB0795w	ATP synthase F1, alpha subunit, putative	mitochondrial	144.591	0.545
PF13_0315	rRNA associated RNA binding protein, putative		143.404	0.737
PFE0770w	conserved Plasmodium protein, unknown function		142.350	0.535
PF14_0745	probable protein, unknown function		141.178	0.389
PF11_0106	apicoplast ribosomal protein L36e precursor, putative	ribosomal	139.670	0.542
PF14_0748	Plasmodium exported protein (PHISTa), unknown function		136.939	0.217
PFC0300c	60S ribosomal protein L7, putative	ribosomal	135.530	0.484
PFE0935c	RNA binding protein Puf1		134.266	0.636
PFC0775w	40S ribosomal protein S11, putative	ribosomal	130.901	0.360
PF13_0070	branched-chain alpha keto-acid dehydrogenase, putative	mitochondrial	130.794	0.567
MAL13P1.231	Pfsec61, putative		130.274	0.374
PF13_0045	40S ribosomal protein S27, putative	ribosomal	129.252	0.470

PFI0110c	serine/threonine protein kinase, FIKK family		128.975	0.104
PF14_0288	cytochrome c oxidase subunit II precursor, putative	mitochondrial	126.224	0.724
PF10_0245	hypothetical protein	apicoplast	124.988	0.456
PF11_0037	Plasmodium exported protein (PHISTb), unknown function		122.768	0.267
MAL8P1.51	secretory complex protein 61 beta subunit		122.171	0.341
PF11_0042	conserved Plasmodium protein, unknown function		121.687	0.123
PFE0940c	secreted ookinete protein, putative		119.616	0.680
PF11_0224	circumsporozoite-related antigen		119.550	0.647
PF14_0198	glycine-tRNA ligase, putative	DNA metabolism, apicoplast	118.481	0.558
PF08_0056	zinc finger protein, putative		117.855	0.766
PFF0865w	histone H3 variant, putative		115.390	0.642
PF14_0373	ubiquinol-cytochrome c reductase iron-sulfur subunit, puta	mitochondrial	114.348	0.425
PFE0225w	3-methyl-2-oxobutanoate dehydrogenase (lipoamide), puta	mitochondrial	114.031	0.471
MAL13P1.2	rifin		113.628	0.135
MAL13P1.132	microfibril-associated protein homologue, putative		112.898	0.802
PFC0535w	60S ribosomal protein L26, putative	ribosomal	110.587	0.538
PF14_0092	conserved Plasmodium membrane protein, unknown function		109.840	0.840
PF14_0690	Histone deactylase, putative	DNA metabolism	109.463	0.805
PF11_0272	ribosomal protein S18, putative	ribosomal	105.831	0.379
PF11_0362	protein phosphatase, putative		105.810	0.638
PF10_0038	ribosomal protein S20e, putative	ribosomal	104.907	0.320
PFA0675w	RESA-like protein with DnaJ domain, putative		104.795	0.740
PF14_0295	ATP-specific succinyl-CoA synthetase beta subunit, putative	mitochondrial	104.736	0.511
PFC1020c	40S ribosomal protein S3A, putative	ribosomal	104.626	0.408
PFA0555c	UMP-CMP kinase, putative	kinase, DNA metabolism, api	103.528	0.474
PFL2525c	Plasmodium exported protein, unknown function		103.160	0.242
PF10_0242	hypothetical protein		102.931	0.741
PFL1400c	hypothetical protein, conserved		101.951	0.701
PFL0010c	rifin		101.110	0.089
PFI1825w	rifin		100.127	0.102
PF14_0272	CPW-WPC family protein		99.430	0.663
PFB0921c	Plasmodium exported protein, unknown function		99.013	0.090
MAL7P1.102	conserved Plasmodium protein, unknown function		98.925	0.627
PFF1455c	CPW-WPC family protein		97.875	0.849

PF11_0197	ankyrin repeat domain protein, putative	apicoplast	97.364	0.510
PFI1095w	conserved Plasmodium protein, unknown function		97.256	0.728
PFL2565w	Plasmodium exported protein (PHISTa), unknown function	apicoplast	97.214	0.143
PF10_0179a	PHF5-like protein, putative		96.560	0.791
PFI1680w	UBX domain, putative		93.795	0.783
MAL8P1.25	conserved Plasmodium protein, unknown function		93.646	0.311
PFA0700c	Plasmodium exported protein (hyp10), unknown function		93.614	0.061
PFI0185w	LCCL domain-containing protein	apicoplast	93.339	0.800
PF11_0503	Plasmodium exported protein (PHISTc), unknown function		91.534	0.134
PFB0075c	Plasmodium exported protein (hyp9), unknown function		91.085	0.093
PF14_0752	Plasmodium exported protein (PHISTa), unknown function		90.573	0.109
PFD1130w	conserved Plasmodium protein, unknown function		90.376	0.378
MAL13P1.92	40S ribosomal protein S15/S19, putative	ribosomal	90.370	0.730
PF11_0113	mitochondrial ribosomal protein L11 precursor, putative	ribosomal, mitochondrial	90.012	0.634
PF11_0260	60S ribosomal protein L35, putative	ribosomal	89.463	0.807
PFD0770c	60S ribosomal protein L15, putative	ribosomal	88.575	0.815
PFL1685w	conserved Plasmodium protein, unknown function		88.412	0.587
PF08_0063	ClpB protein, putative	peptidase activity, apicoplast	88.178	0.631
PFF1170w	conserved Plasmodium protein, unknown function		87.658	0.572
PF11_0455a	conserved Plasmodium protein, unknown function	ribosomal	87.440	0.611
PF13_0358	mitochondrial import inner membrane translocase, putative	mitochondrial	87.068	0.630
PF10_0052	conserved Plasmodium protein, unknown function		86.235	0.761
PFA0225w	4-hydroxy-3-methylbut-2-enyl diphosphate reductase	apicoplast	85.883	0.769
MAL13P1.130	glideosome associated protein with multiple membrane spans 1		85.565	0.712
PFD0470c	replication protein A large subunit	DNA replicaton	84.961	0.558
PF14_0598	glyceraldehyde-3-phosphate dehydrogenase, putative		84.274	0.653
PFD0185c	hypothetical protein, conserved		83.966	0.677
PF08_0024	conserved Plasmodium protein, unknown function		83.574	0.481
PF08_0139	rifin		82.952	0.102
PFC0185w	membrane skeletal protein IMC1-related		82.867	0.717
MAL13P1.270	proteasome subunit, putative	proteasome	82.441	0.541
PF08_0081	hypothetical protein		81.604	0.755
PFF0130c	conserved Plasmodium protein, unknown function		81.482	0.799
PFC0475c	phosphatidylinositol 3- and 4-kinase, putative	kinase	81.205	0.620

PF11_0464	serine/threonine protein kinase, putative	kinase, apicoplast	80.969	0.825
PFB0680w	rhoptry neck protein 6		80.554	0.528
PFE0810c	40S ribosomal subunit protein S14, putative	ribosomal	80.528	0.608
PF14_0068	fibrillarin, putative	mitochondrial, DNA metabol	79.993	0.728
PFD1085w	conserved Plasmodium protein, unknown function		79.371	0.775
PF14_0695	DNA-directed RNA polymerase, alpha subunit, putative	DNA metabolism, apicoplast	79.202	0.721
PF14_0391	60S ribosomal protein L1, putative	ribosomal	78.890	0.487
PFE0185c	60S ribosomal subunit protein L31, putative	ribosomal	78.785	0.480
MAL13P1.168	protein tyrosine phosphatase, putative		78.569	0.740
MAL13P1.129	hypothetical protein		78.424	0.628
MAL7P1.125	hypothetical protein		77.935	0.835
PFC0495w	aspartyl protease, putative	peptidase activity	77.342	0.891
PF14_0425	fructose-bisphosphate aldolase, putative		77.203	0.756
PFA0480w	phenylalanyl-tRNA synthetase beta chain, putative	DNA metabolism	77.031	0.576
MAL7P1.208	rhoptry-associated membrane antigen		76.888	0.562
PFI0865w	XPA binding protein 1, putative		76.706	0.562
PFE0070w	interspersed repeat antigen		76.687	0.591
MAL8P1.146	filament assembling protein, putative		76.483	0.627
PF14_0744	Plasmodium exported protein, unknown function		76.455	0.295
PFF0305c	ubiquitin conjugating enzyme E2, putative		76.453	0.754
PFE0675c	deoxyribodipyrimidine photolyase (photoreactivating enzyr	DNA metabolism	76.215	0.780
PFD0025w	rifin		76.017	0.176
PFL1695c	conserved Plasmodium protein, unknown function		75.764	0.851
PF14_0024	conserved Plasmodium protein, unknown function		75.535	0.884
PFD0700c	RNA binding protein, putative	DNA metabolism	74.967	0.708
MAL8P1.17	disulfide isomerase precursor, putative		74.611	0.812
PF10_0334	flavoprotein subunit of succinate dehydrogenase	mitochondrial	74.071	0.629
PFI1435w	RNA binding function, putative		73.828	0.627
PFL0585w	polyubiquitin		73.785	0.609
PF14_0486	elongation factor 2, putative		73.603	0.808
PFL0110c	mitochondrial phosphate carrier protein, PfmpC	mitochondrial	73.573	0.790
PFC0805w	DNA-directed RNA polymerase II, putative	DNA metabolism	73.025	0.590
PF11_0292	cochaperone prefoldin complex subunit, putative		72.016	0.764
PFI1750c	Plasmodium exported protein (hyp11), unknown function		71.883	0.718

PFC0295c	40S ribosomal protein S12, putative	ribosomal	71.516	0.423
PF07_0054	histone H2B variant, putative	DNA metabolism	71.412	0.762
PFA0505c	DNA-directed RNA polymerase ii 15.1 kDa polypeptide, putative		71.026	0.780
PF13_0248	transmission blocking target antigen precursor, Pbs47		70.989	0.838
PFD0385w	conserved Plasmodium protein, unknown function		70.714	0.763
PFC0990c	conserved Plasmodium protein, unknown function		70.544	0.797
PFC0290w	40S ribosomal protein S23, putative	ribosomal	70.073	0.816
PF08_0097	hypothetical protein		69.937	0.752
PF13_0168	CPW-WPC family protein	apicoplast	69.566	0.784
PFI0705w	conserved hypothetical protein		69.470	0.630
PFL0305c	IMP-specific 5'-nucleotidase, putative,haloacid dehalogenase hydrolase, putative		69.464	0.759
PF11_0454	40S ribosomal protein S21e, putative	ribosomal	69.211	0.492
PF14_0231	60S ribosomal protein L7-3, putative	ribosomal	68.793	0.573
PF14_0571	conserved Plasmodium membrane protein, unknown function		68.746	0.794
PFF0665c	syntaxin binding protein, putative		68.603	0.725
PF14_0538	conserved Plasmodium protein, unknown function		68.154	0.778
PFC0845c	ubiquitin--protein ligase, putative	apicoplast	68.100	0.869
PFD0885c	conserved Plasmodium protein, unknown function		67.927	0.769
PFL0285w	targeted glyoxalase II	apicoplast	67.588	0.746
PFI1800w	lysophospholipase, putative		67.460	0.134
PF11_0258	co-chaperone GrpE, putative	mitochondrial	67.207	0.659
PFF1575w	rifin		67.111	0.130
PFA0635c	Plasmodium exported protein (hyp1), unknown function		67.075	0.305
PFF0975c	conserved Plasmodium protein, unknown function	attachment of GPI anchor to protei	66.518	0.583
PFD1035w	steroid dehydrogenase kik-i, putative		66.292	0.859
PFE0075c	rhoptry-associated protein, putative		66.242	0.346
MAL13P1.40	conserved Plasmodium protein, unknown function	kinase	66.094	0.774
PFF1330c	mitochondrial import inner membrane translocase subunit,	mitochondrial	65.514	0.838
PF11_0084	conserved Plasmodium protein, unknown function		65.394	0.657
PF13_0316	40S ribosomal protein S13, putative	ribosomal	65.088	0.865
PFI0680c	arginyl-tRNA synthetase, putative	apicoplast	64.658	0.721
PFC0955w	ATP-dependent RNA helicase, putative		64.457	0.778

Enriched in ΔPuf2 (118 genes)

Gende ID	Annotation	Functional Category	t-score	Fold change in expression
PF14_0337	conserved Plasmodium protein, unknown function		-57.734	1.191
PFC0710w-a	inorganic pyrophosphatase, putative(-a)?		-57.936	1.118
PF14_0784	ribosome biogenesis protein, NOP10-like		-57.979	1.226
PF14_0067	LCCL domain-containing protein		-58.028	1.205
PFE0230w	conserved Plasmodium protein, unknown function		-58.247	1.504
PF14_0100	cytidine triphosphate synthetase	DNA metabolism	-59.186	1.916
PFE0570w	RNA pseudouridylate synthase, putative		-59.330	1.269
PFI1525w	conserved Plasmodium protein, unknown function		-59.427	1.212
PFC0940c	conserved Plasmodium protein, unknown function		-59.575	1.866
PF11_0147	mitogen-activated protein kinase 2,	Pr kinase	-59.764	1.899
PF11_0199	conserved Plasmodium protein, unknown function	translation / seryl-tRNA aminoacyla	-59.805	1.135
PF13_0212	conserved Plasmodium protein, unknown function	translation / seryl-tRNA aminoacyla	-60.701	1.505
PF13_0254	conserved Plasmodium membrane protein, unknown function		-60.823	1.321
PF11_0372	conserved hypothetical protein	apicoplast	-61.104	1.217
PFF1010c	DnaJ protein, putative		-61.718	1.147
PF10_0285	conserved Plasmodium protein, unknown function		-61.792	1.360
MAL13P1.202	conserved Plasmodium protein, unknown function		-62.425	1.505
PF10_0264	40S ribosomal protein S2B, putative	ribosomal	-62.579	1.381
PF14_0705	conserved hypothetical protein		-62.757	1.509
PF10_0171	conserved Plasmodium protein, unknown function		-62.883	1.174
PFI0440w	conserved Plasmodium protein, unknown function		-62.989	1.228
PF11_0320	RNA-binding protein s1, putative		-63.277	1.373
PFL2100w	ubiquitin conjugating enzyme E2, putative		-63.725	1.309
PF14_0613	conserved Plasmodium protein, unknown function		-63.913	1.303
PF11_0204	conserved Plasmodium protein, unknown function		-64.029	1.802
MAL7P1.29	conserved Plasmodium protein, unknown function		-65.289	1.216
PFA0425c	conserved Plasmodium protein, unknown function	DNA replication	-65.933	1.258
PFB0227c	conserved Plasmodium protein, unknown function		-66.146	1.504
PFF0215w	conserved Plasmodium protein, unknown function	apicoplast	-66.203	1.236
PFE1095w	conserved Plasmodium protein, unknown function		-66.503	1.361
MAL8P1.60	conserved Plasmodium protein, unknown function		-66.943	1.510

PF14_0522*	conserved Plasmodium protein, unknown function		-67.266	1.597
PF14_0463	chloroquine resistance marker protein		-67.603	1.585
PF13_0050	HORMA domain protein, putative		-67.789	1.297
PFB0405w	transmission-blocking target antigen s230 precursor		-67.880	1.505
PFB0655c	conserved Plasmodium protein, unknown function		-68.050	1.396
PF11_0422	conserved Plasmodium protein, unknown function		-68.393	1.174
PF14_0492	protein phosphatase 2b regulatory subunit, putative		-68.531	1.832
PF10_0076	conserved Plasmodium protein, unknown function		-69.500	1.615
PFI1570c	M18 aspartyl aminopeptidase		-69.670	1.257
PF14_0363	metacaspase-like protein		-69.689	1.405
MAL13P1.221	aspartate carbamoyltransferase	apicoplast	-69.926	1.381
PF13_0087	1-methyladenosine tRNA methyltransferase subunit, putative		-70.563	1.293
MAL8P1.77	conserved hypothetical protein		-71.358	2.338
PF13_0058	conserved hypothetical protein		-71.597	1.119
PFL0325w	Tat binding protein 1(TBP-1)-interacting protein, putative		-71.863	1.398
PF10_0104	dolichyl-phosphate-mannose protein mannosyltransferase, putative		-72.286	1.200
PFI1210w	conserved Plasmodium protein, unknown function		-72.404	1.136
PF07_0003	rifin		-72.657	1.720
PF14_0291	conserved hypothetical protein		-72.996	1.276
PF08_0026	conserved protein, unknown function		-73.155	1.243
PFL0365c	hypothetical protein, conserved	apicoplast	-73.417	1.235
PFI1270w	conserved Plasmodium protein, unknown function		-74.486	1.346
PF08_0128	conserved Plasmodium protein, unknown function		-76.370	1.393
PFI1465w	procollagen lysine 5-dioxygenase, putative		-78.134	2.075
PFA0290w	DNA binding protein, putative		-79.353	1.206
PF14_0450	conserved Plasmodium protein, unknown function		-79.720	1.172
PF07_0061	conserved Plasmodium protein, unknown function		-79.943	1.549
PFF0920c	conserved Plasmodium protein, unknown function		-80.462	1.523
PF10_0178	conserved hypothetical protein		-81.406	1.537
PFE0320w	conserved Plasmodium protein, unknown function		-81.901	1.340
PFL0445w	conserved Plasmodium protein, unknown function		-82.074	1.858
PFE1173c	outer arm dynein lc3, putative	dynein	-82.300	1.601
PFI1230c	conserved Plasmodium protein, unknown function		-82.870	1.209
PF11_0481	tubulin-tyrosine ligase, putative		-83.079	1.371

PFL1015w	conserved Plasmodium protein, unknown function		-83.223	1.212
PFI0775w	glycolipid transfer protein, putative		-83.657	1.211
PFD0580c	conserved Plasmodium protein, unknown function		-83.754	1.401
PF11_0477	CCAAT-box DNA binding protein subunit B		-83.858	1.505
PF14_0143	atypical protein kinase, ABC-1 family, putative	kinase	-84.073	1.276
PFL2320w*	conserved Plasmodium protein, unknown function	apicoplast	-86.005	1.632
MAL8P1.109	protein phosphatase, putative		-86.903	1.987
PFL1095c	conserved Plasmodium protein, unknown function		-87.458	1.232
PF11_0412	Vacuolar ATP synthase subunit F, putative	DNA metabolism	-87.750	2.080
PFI1085w	ubiquitin-like protein, putative		-87.756	1.328
PFL1200c	splicing factor 3b subunit, putative	mitochondrial	-89.605	1.285
PFD1020c	rifin		-90.626	2.398
PF08_0122	conserved Plasmodium protein, unknown function		-93.191	1.206
PF07_0087	hypothetical protein, conserved	apicoplast	-93.342	1.862
PF13_0021	small heat shock protein, putative		-93.756	1.505
PFI1350c	dynein light chain, putative	dynein	-94.535	1.416
PF07_0030	heat shock protein 86 family protein		-97.049	1.504
PF13_0165	conserved hypothetical protein		-97.231	1.207
PF11_0148	dynein light chain type 2, putative	dynein	-98.811	1.988
PFB0895c	replication factor C subunit 1, putative	DNA replicaton, DNA metabo	-100.146	3.967
MAL8P1.7	conserved Plasmodium protein, unknown function		-100.424	1.504
PF10_0226	hypothetical protein, conserved		-102.081	1.442
PF13_0310	small subunit rRNA processing factor, putative		-106.521	1.505
PF10_0115	QF122 antigen		-107.422	1.218
PF11_0087*	Rad51 homolog, putative	DNA metabolism	-109.511	2.165
MAL13P1.262	conserved Plasmodium protein, unknown function	apicoplast	-111.561	1.599
PFD0195c	conserved Plasmodium protein, unknown function		-114.173	1.228
PFE0415w	transcription factor iib, putative	DNA metabolism	-114.840	2.615
PF11_0458	conserved Plasmodium protein, unknown function		-114.960	1.402
PF14_0601	replication factor C3	DNA replicaton	-115.133	2.092
PF10_0279	conserved Plasmodium protein, unknown function		-117.810	1.276
PFA0360c	hypothetical protein conserved in P. falciparum		-119.460	1.404
MAL13P1.96	chromosome segregation protein, putative	dynein, DNA replicaton	-121.823	1.972
PF14_0805	conserved Plasmodium protein, unknown function		-123.113	1.240

PF10_0303*	25 kDa ookinete surface antigen precursor (pfs25)		-130.608	1.541
MAL8P1.46	outer arm dynein light chain 2	dynein	-130.905	1.505
PFD0993c	conserved Plasmodium protein, unknown function		-132.233	1.515
PF14_0543	signal peptide peptidase, mSPP	peptidase activity, apicoplast	-133.641	1.341
PF10_0195a	flagellar outer arm dynein-associated protein, putative		-134.978	1.350
PF08_0017	conserved Plasmodium protein, unknown function		-135.510	1.330
PF11_0117	replication factor C subunit 5, putative	DNA replicaton	-141.028	1.869
PF07_0042	conserved Plasmodium protein, unknown function		-151.302	1.429
PF13_0095	DNA replication licensing factor mcm4-related	DNA replicaton	-163.736	1.623
PFD0590c	DNA polymerase alpha	DNA polymerase, DNA replic	-165.651	1.797
MAL13P1.195*	CPW-WPC family protein		-181.628	1.804
PF14_0352	ribonucleoside-diphosphate reductase, large subunit		-183.896	2.158
PF13_0353	NADH-cytochrome b5 reductase, putative		-235.362	1.505
PFC0260w	hypothetical protein, conserved	apicoplast	-236.019	1.829
PF13_0121	dihydrolipoamide succinyltransferase, putative	mitochondrial	-295.431	2.514
PF11_0057	conserved Plasmodium protein, unknown function	dynein	-338.667	7.778
PF10_0302*	ookinete surface protein Pos28-1, putative		-359.195	1.699
PF08_0004	hypothetical protein, conserved	apicoplast	-374.086	1.978
PF14_0030	hypothetical protein	dynein, apicoplast	-467.444	1.619

Table S1 C. Up- and -down-regulated transcripts in both stage III and stage V gametocyte between 3D7 wildtype and ΔPfPuf2 line.

1. Gene ID columns highlighted in green color are genes also identified by SAM; Gene ID with * and annotation columns filled with red color are the TR genes

2. t score differing for more than 2 standard deviations from the mean t-statistic value or fold change larger than 2 between average transcripts of 3d7 and ΔPfPuf2 are colored by dark blue, while 1.5 fold change by light blue

Gene ID[1]	Annotation	Functional Category	Stage III t-score[2]	Stage III Fold change in expression	Stage V t-score	Stage V Fold change in expression
	**Enriched in 3D7 (59 genes)**					
MAL13P1.480	histidine-rich protein III (hrp3)		225.355	0.046	731.429	0.010
MAL13P1.164	elongation factor tu, putative	mitochondrial	200.544	0.638	551.273	0.355
PFI1735c	ring-exported protein 1		129.721	0.098	548.061	0.031
PFC0400w	60S acidic ribosomal protein P2, putative	ribosomal	565.891	0.611	534.206	0.544
PF11_0043	60S acidic ribosomal protein p1, putative	ribosomal	357.038	0.706	494.059	0.672
PF13_0346	hypothetical protein	ribosomal	397.126	0.460	435.628	0.405
PF08_0126	DNA repair protein rad54, putative	DNA metabolism	154.845	0.545	366.489	0.524
PFB0295w	adenylosuccinate lyase, putative	DNA metabolism	181.667	0.429	334.119	0.392
PFE0845c	60S ribosomal subunit protein L8, putative	ribosomal	101.299	0.588	326.919	0.427
MAL7P1.100	serine/threonine protein kinase 2, putative	kinase	263.560	0.506	325.339	0.563
PF07_0079	60S ribosomal protein L11a, putative	ribosomal	115.857	0.585	286.379	0.123
PFE1595c	Plasmodium exported protein (PHISTc)		145.678	0.279	266.493	0.150
PF13_0032	hydrolase, putative	apicoplast	174.322	0.435	245.898	0.604
PF13_0268	ribosomal protein L17, putative	ribosomal	125.106	0.389	240.857	0.427
PFF0860c	histone H2A	histone	134.604	0.675	235.078	0.722
PFB0455w	ribosomal L37ae protein, putative	ribosomal	188.349	0.474	234.657	0.556
PF10_0144	prohibitin, putative	mitochondrial	250.821	0.542	234.071	0.612
PF08_0076	40S ribosomal protein S16, putative	ribosomal	224.179	0.359	231.912	0.320
PFB0115w	conserved Plasmodium protein		123.929	0.106	219.129	0.059
PF13_0242	isocitrate dehydrogenase (NADP), putative	mitochondrial	224.262	0.558	217.864	0.517
PFE0495w	conserved hypothetical protein	apicoplast	179.437	0.503	207.150	0.551
PF10_0366	ADP/ATP transporter on adenylate translocase	mitochondrial	266.689	0.634	205.855	0.390
PFE0480c	hypothetical protein	apicoplast	120.209	0.604	193.153	0.710
PF14_0597	cytochrome c1 precursor, putative	mitochondrial	150.525	0.400	189.747	0.500
PF13_0011	P. falciparum gamete antigen 27/25 (Pfs27/25)		128.665	0.649	177.426	0.635

PF10_0345	merozoite surface protein 3 (msp3)		79.312	0.158	177.381	0.175
PFD1055w	ribosomal protein S19s, putative	ribosomal	153.233	0.396	162.478	0.616
PFI1625c	organelle processing peptidase, putative	mitochondrial	222.403	0.421	160.941	0.446
PF11_0351	heat shock protein hsp70 homologue	mitochondrial	133.320	0.477	158.346	0.593
PF14_0240	ribosomal protein L21e, putative	ribosomal	144.616	0.500	157.960	0.470
PF13_0129	ribosomal protein L6 homologue, putative	ribosomal	126.605	0.611	154.435	0.669
PF14_0083	ribosomal protein S8e, putative	ribosomal	209.803	0.481	153.697	0.486
PFB0795w	ATP synthase F1, alpha subunit, putative	mitochondrial	147.171	0.469	144.591	0.545
PFE0770w	hypothetical protein		99.075	0.544	142.350	0.535
PFC0300c	60S ribosomal protein L7, putative	ribosomal	208.074	0.374	135.530	0.484
PF13_0070	branched-chain alpha keto-acid dehydrogenase	mitochondrial	168.962	0.399	130.794	0.567
PFI0110c	serine/threonine protein kinase, FIKK family	kinase	78.044	0.225	128.975	0.104
PF10_0245	glucosamine--fructose-6-phosphate aminotransferase	apicoplast	159.773	0.368	124.988	0.456
MAL8P1.51	secretory complex protein 61 beta subunit		87.610	0.314	122.171	0.341
PF11_0224	circumsporozoite-related antigen		86.889	0.705	119.550	0.647
PF14_0198	glycine --tRNA ligase, putative	apicoplast	85.230	0.550	118.481	0.558
PF14_0373	ubiquinol cytochrome c oxidoreductase, putative	mitochondrial	168.015	0.467	114.348	0.425
PFC0535w	60S ribosomal protein L26, putative	ribosomal	184.051	0.504	110.587	0.538
PF14_0092	conserved Plasmodium protein		81.912	0.745	109.840	0.840
MAL8P1.25	conserved hypothetical protein		74.391	0.360	93.646	0.311
PFA0700c	Plasmodium exported protein (hyp10)		73.054	0.206	93.614	0.061
PF11_0260	ribosomal protein L35, putative	ribosomal	198.529	0.519	89.463	0.807
PF08_0063	conserved hypothetical protein	apicoplast	288.279	0.276	88.178	0.631
PFF1170w	conserved hypothetical protein		91.206	0.617	87.658	0.572
PF13_0358	mitochondrial import inner membrane translocase	mitochondrial	185.173	0.397	87.068	0.630
PFA0225w	LytB protein	apicoplast	165.564	0.442	85.883	0.769
PFD0470c	replication factor a protein, putative	DNA metabolism	126.265	0.506	84.961	0.558
MAL13P1.270	proteosome subunit, putative	proteosome	181.838	0.520	82.441	0.541
PFE0810c	40S ribosomal subunit protein S14, putative	ribosomal	170.750	0.419	80.528	0.608
PF07_0054	histone H2B variant, putative	histone	99.657	0.718	71.412	0.762
PFC0290w	40S ribosomal protein S23, putative	ribosomal	207.806	0.595	70.073	0.816
PF08_0097	hypothetical protein		100.207	0.450	69.937	0.752
PF14_0231	ribosomal protein L7a, putative	ribosomal	178.213	0.470	68.793	0.573
PFC0845c	ubiquitin--protein ligase, putative	proteosome	79.312	0.158	68.100	0.869

Enriched in ΔPuf2 (18 genes)

PFI0440w	hypothetical protein		-123.827	1.481	-62.989	1.228
PFB0405w	Pfs230		-156.377	2.534	-67.880	1.461
PF14_0492	protein phosphatase 2b regulatory subunit, putative		-117.362	1.933	-68.531	1.832
PFL0445w	conserved Plasmodium protein		-99.198	2.394	-82.074	1.858
PF07_0087	conserved hypothetical protein	apicoplast	-184.698	2.907	-93.342	1.862
PFI1350c	dynein light chain, putative	axoneme/flagella	-148.943	1.600	-94.535	1.416
PF11_0148	dynein light chain type 2, putative	axoneme/flagella	-223.922	2.078	-98.811	1.988
PFB0895c	replication factor C subunit 1, putative	DNA metabolism	-103.191	2.549	-100.146	3.967
PFE0415w	transcription factor iib, putative	DNA metabolism	-179.296	1.959	-114.840	2.615
PF14_0601	replication factor C3, putative	DNA metabolism	-190.368	2.909	-115.133	2.092
MAL13P1.96	chromosome segregation protein, putative	DNA metabolism	-152.526	1.873	-121.823	1.972
PF10_0303*	25kDa ookinete surface antigen, P25	ookinete surface antig	-260.851	8.147	-130.608	1.541
PF13_0095	minchromosome maintenance (MCM) complex subunit	DNA metabolism	-205.172	2.677	-163.736	1.623
MAL13P1.195*	CPW-WPC family protein		-111.774	1.590	-181.628	1.804
PFC0260w	P-loop containing nucleoside triphospahte hydrolase	DNA metabolism	-212.287	2.439	-236.019	1.829
PF11_0057	radial spoke head protein	axoneme/flagella	-305.183	2.026	-338.667	7.778
PF10_0302*	28kDa ookinete surface antigen, P28	ookinete surface prote	-156.553	1.716	-359.195	1.699
PF14_0030	hypothetical protein	axoneme/flagella	-217.427	1.406	-467.444	1.619

Table S1D. Shared transcripts between up-regulated (>2 fold) in ΔPfPuf2 gametocytes (Stage III and V) and down-regulated in both ΔDOZ1 and ΔCITH in P. berghei gametocytes.

Pf Gene ID	Pb Gene ID	Annotation
**Stage III**		
MAL13P1.195*	PB001530.02.0	CPW-WPC family protein
MAL7P1.74	PB001203.02.0	conserved Plasmodium protein, unknown function
PF07_0029	PB001532.02.0	heat shock protein 86
PF10_0302*	PB000865.00.0	28 kDa ookinete surface protein
PF10_0303*	PB000266.01.0	25 kDa ookinete surface antigen precursor (pfs25)
PF11_0160	PB000981.00.0	conserved Plasmodium protein, unknown function
PF13_0226*	PB000249.03.0	conserved Plasmodium protein, unknown function
PFC0495w*	PB000864.03.0	plasmepsin VI, ASP
PFE0530w	PB403516.00.0	conserved Plasmodium protein, unknown function
PFF1035w	PB001166.02.0	Pfs77 protein
PFI0185w	PB000955.03.0	conserved Plasmodium protein, unknown function
**Stage V**		
MAL13P1.195*	PB001530.02.0	CPW-WPC family protein
PF10_0115	PB000924.03.0	QF122 antigen
PF10_0302*	PB000865.00.0	28 kDa ookinete surface protein
PF10_0303*	PB000266.01.0	25 kDa ookinete surface antigen precursor (pfs25)
PF11_0087*	PB001059.00.0	Rad51 homolog, putative
PF14_0143	PB000717.03.0	Atypical protein kinase, ABC-1 family, putative
PFI0775w	PB000907.00.0	glycolipid transfer protein, putative
PFL2320w*	PB000245.02.0	conserved Plasmodium protein, unknown function

Asterisks (*) indicate TR transcripts identified in P. bergher gametocytes.
