# Supplementary material for: Puf Mediates Translation Repression of Transmission-Blocking Vaccine Candidates in Malaria Parasites
Source: PLoS Pathog. 2013 Apr 18;9(4):e1003268. doi: 10.1371/journal.ppat.1003268 (PMC3630172; doi:10.1371/journal.ppat.1003268)

Table S2. Relative *luciferase* mRNA levels and luciferase activities in gametocytes of stably transfected 3D7-AttB parasites.

Constructs	***Stage III***			***Stage V***		
	***Luc* mRNA levels**		**Luc activity**	***Luc* mRNA levels**		**Luc activity**
	Luc Primers	3' or 5' UTR Primers		Luc Primers	3' or 5' UTR Primers	
Hsp86-LUC-pDT3'	12.86	10.37(3')	341.17	16.99	13.92(3')	201.27
Hsp86-LUC-Pfs25 3'	15.99	13.28(3')	263.60	18.98	16.73(3')	126.20
Hsp86-LUC-Pfs28 3'	10.79	9.26(3')	3.20	13.67	11.73(3')	1.56
Pfs47-LUC-pDT3'	2.16	1.98(3')	69.03	2.42	2.23(3')	60.66
Pfs47-LUC-Pfs25 3'	2.85	2.48(3')	52.01	3.26	2.96(3')	51.45
Pfs47-LUC-Pfs28 3'	1.70	1.85(3')	1.03	1.716	1.53(3')	0.44
α-TubII-LUC-pDT3'	8.62	7.83(3')	244.23	10.98	11.03(3')	182.90
α-TubII-LUC-Pfs25 3'	9.32	10.09(3')	211.27	12.81	11.93(3')	103.67
α-TubII-LUC-Pfs28 3'	6.75	7.25(3')	6.22	8.18	7.47(3')	2.62
Pfs25-LUC-pDT3'	4.02	4.81(5')<br>3.85 (3)	4.16	5.36	6.29(5')<br>4.75(3)	1.39
Pfs25-LUC-Pfs25 3'	5.26	4.27(5')<br>4.81(3')	3.37	7.38	7.15(5')<br>6.04(3')	1.37
Pfs28-LUC-pDT3'	1.09	1.65(5')<br>1.36(3')	60.58	1.28	2.13(5')<br>1.57(3')	38.01
Pfs28-LUC-Pfs28 3'	2.47	2.13(5')<br>1.85(3')	1.24	3.57	3.16(5')<br>2.84(3')	0.73
Hsp86-LUC-Pfs28 3'UTR-pDT3'	9.97	10.28(3')	14.87	14.03	12.21(3')	4.65
Pfs47-LUC-Pfs28 3' UTR-pDT3'	1.50	1.39(3')	1.15	1.64	1.52(3')	0.57
α-TubII-LUC-Pfs28 3' UTR-pDT3'	5.56	6.57(3')	1.91	7.16	6.37(3')	1.28
Hsp86-LUC-Pfs25 5'UTR-pDT3'	9.11	8.72(3')	3.20	12.46	10.93(3')	2.04
Pfs47-LUC-Pfs25 5'UTR-pDT3'	1.68	1.49(3')	0.83	1.56	1.68(3')	0.25
α-TubII-LUC-Pfs25 5'UTR-pDT3'	6.39	5.96(3')	7.62	5.83	6.17(3')	7.83
Hsp86-LUC-Pfs25 5'UTR-r-pDT3'	13.92	11.38(3')	295.43	15.22	13.84(3')	264.76

Note: All parasite lines were generated with the mycobacteriophage Bxb1 integrase so that only one copy of the construct is integrated into the parasite genome. Gametocytes were harvested at stage III and stage V. The parasites were split into two halves. One half was lysed and equal amount (100 µg) of gametocyte lysates was used for measuring luciferase activity (Luc) by using the Luciferase Assay System (Promega). Another half was used for RNA purification by TRIzol. The extracted RNA was used for cDNA synthesis and real-time RT-PCR as previously described (Miao et al., 2006) with primers designed for Luc transcripts and an internal reference transcript of *PF07_0073*. The primers were designed to amplify C terminal end of Luc ORF, UTRs of *Pfs25*, *Pfs28*, and pDT3' and the PCR fragments are shown in the scheme in the right panel. In order to avoid to amplifying the endogenous UTRs of Pfs25 and Pfs28, one primer for amplifying the UTRs is located in Luc ORF.

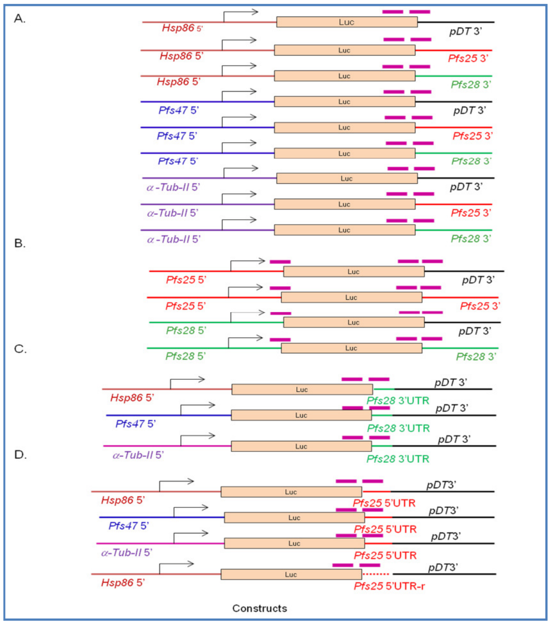

Supplement: Table S2 — Relative luciferase mRNA levels and luciferase activities in gametocytes of stably transfected 3D7-AttB parasites. (PDF) [file ppat.1003268.s005.pdf]
