# Supplementary material for: Puf Mediates Translation Repression of Transmission-Blocking Vaccine Candidates in Malaria Parasites
Source: PLoS Pathog. 2013 Apr 18;9(4):e1003268. doi: 10.1371/journal.ppat.1003268 (PMC3630172; doi:10.1371/journal.ppat.1003268)
Supplement: Table S3 — Primers for PCR, mutagenesis, RACE, and in vitro transcription. (DOCX) [file ppat.1003268.s006.docx]

**Table S3.** **Primers for PCR, mutagenesis, RACE, and *in vitro* transcription**

| NName | Sequence | Description |
| --- | --- | --- |
| Pfs25 5' FR-F | gcactagtACAACTCATAACAAGCTTGAT | PCR of Pfs25 5' flanking region |
| Pfs25 5' FR-R | gaagatctGAATAAAAAAATATAAGGTATGAG |  |
| Pfs25 3' FR-F | ccgctcgagcctaggTTTATACTATTTTCAGTATGCTT | PCR of Pfs25 3' flanking region |
| Pfs25 3' FR-R | ataagaatccggccgccTGTCCAAATGCATGAACAAGTC |  |
| Pfs28 5' FR-F | ggactagtCCATGAACTCTAATAAAATGATATC | PCR of Pfs28 5' flanking region |
| Pfs28 5' FR-R | gaagatctGTATAAAAAACTAAAAAATATAAAATAATAAG |  |
| Pfs28 3' FR-F | ccgctcgagcctaggGTCATATGATTTGCATCTTATTTC | PCR of Pfs28 3' flanking region |
| Pfs28 3' FR-R | ataagaatgcggccgcCATACGCGTCCTGGGGC |  |
| PFL2320w 5' FR-F | ggactagtGAACATCCTTTGTATATAGGTG | PCR of PFL2320w 5' flanking region |
| PFL2320w 5' FR-R | gaagatctTCTTAAAAGCTGAAAATTTTAGA |  |
| PFL2320w 3' FR-F | ccgctcgagcctaggATGAATATATACATATATATAGATA | PCR of PFL2320w 3' flanking region |
| PFL2320w 3' FR-F | ataagaatgcggccgcGTCACATTAGCAAGCGCAAG |  |
| PmVI 5' FR-F | gcactagtACATTTCCTTCAAGTAAGC | PCR of PmVI 5' flanking region |
| PmVI 5' FR-R | gaagatctATATAAAATTTCTGGAATAAAAAC |  |
| PmVI 3' FR-F | ccgctcgagcctaggCTAATTGAAGCTAATCATAACTT | PCR of PmVI 3' flanking region |
| PmVI 3' FR-R | ataagaatgcggccgcgTAGACAAGGTACTAAAATATCTG |  |
| α-Tub II 5' FR-F | ggactagtGTACACATGTAAAATACCTC | PCR of α tubulin II 5' flanking region |
| α-Tub II 5' FR-R | gaagatctCGTTCGTAAAACGTTAGATAA |  |
| Pfs47 5' FR-F | ggactagtCATTCCTAACACATTATGTGT | PCR amplification of Pfs47 5' flanking region |
| Pfs47 5' FR-R | gaagatctAAAATAAACAAAAAAATAAACTCAAT |  |
| Luc-F | GCAACTGCATAAGGCTATGCCG | RT-PCR for luciferase |
| Luc-R | CACTGCATACGACGATTCTGTG |  |
| GFP-F | CTGTCAGTGGAGAGGGTGAAG | RT-PCR for GFP |
| GFP-R | GACAAGTGTTGGCCATGGAAC |  |
| Pfs25-F | GTTCAAAAGATGGAGAAACC | RT-PCR for Pfs25 |
| Pfs25-R | GCAGTACATATAGAGCTTTC |  |
| Pfs28-F | GAAGTGTGACATTAGAAGG | RT-PCR for Pfs28 |
| Pfs28-R | GAGCATACAATCAGAACGTG |  |
| PFL2320w-F | GAATAGATTGAGAAATTTAATGTC | RT-PCR for PFL2320w |
| PFL2320w-R | GAAATGTATCCGCTTAATTTTTCCC |  |
| PmVI-F | GAAGAAGGGGACACTGAG | RT-PCR for PmVI t |
| PmVI-R | TTATGATTAGCTTCAATTAGACC |  |
| PF14_0508-F | CCACTAATGTCTATGACAAACG | RT-PCR for PF14_0508 |
| PF14_0508-R | CATATGAAGAACATATCAAGCA |  |
| PF07_0073-F | CACGGAAAATAAATATCATGTTC | RT-PCR for PF07_0073 |
| PF07_0073-R | CCATAAAGGGAATAAAATCC |  |
| Pfs28M-F | GAAATGTTCTTTTCCAATTATATTTTG | Site directed mutagenesis at Pfs28 3' UTR |
| Pfs28M-R | CAAAATATAATTGGAAAAGAACATTTC |  |
| Pfs25M-F | TTCTCATTTCCTAATAAATTGTTG | Site directed mutagenesis at Pfs25 5' UTR |
| Pfs25M-R | CAACAATTTATTAGGAAATGAGAA |  |
| Pfs25 5' UTR-F | ccgctcgagATTTTGTTAATTTATAAAATATTATTATGTCG | PCR of Pfs25 5' UTR and RT-PCR of Pfs25 5' UTR –luciferase ORF |
| Pfs25 5' UTR-R  Luc R | ccgctcgagTTTTAAAAGAATAAAAAAATATAAGGTATGAG  CAAGCTTACTTAGATCGCAGATC |  |
| Pfs25 3' UTR-F  Luc F | ccgctcgagTTTATACTATTTTCAGTATGCTT  GGAAAGATCGCCGTGTAATTC | PCR of Pfs25 3' UTR and RT-PCR of Pfs25 3' UTR –luciferase ORF |
| Pfs25 3' UTR-R | ccgctcgagCTGAATTTACCAAATTTTAATATTAG |  |
| Pfs28 5' UTR-F | ccgctcgagCATTAAAAATAAAATTAAAATAAATAAATAACG | PCR of Pfs28 5' UTR |
| Pfs28 5' UTR-R | ccgctcgaGTATAAAAAACTAAAAAATATAAAATAATAAG |  |
| Pfs28 3' UTR-F | ccgctcgagTCATATGATTTGCATCTTATTTC | PCR of Pfs28 3' UTR |
| Pfs28 3' UTR-R | ccgctcgagAGAAAACAATATGTTTTTTGCACAAC |  |
| Pfs25 RNA1-F | ccgctcgagTTCTCATTTGTTAATAAATTGTT | DNA corresponding to Pfs25 5' RNA1 |
| Pfs25 RNA1-R | ccgctcgagTTGTTTTCACAACAATTTATTAAC |  |
| Pfs25M RNA1-F | ccgctcgagTTCTCATTTCCTAATAAATTGTT | DNA corresponding to Pfs25 5' RNA1 with mutation |
| Pfs25M RNA1-R | ccgctcgagTTGTTTTCACAACAATTTATTAGG |  |
| Pfs28 RNA1-F | ccgctcgagGAAATGTTCTTTTGTAATTATATTTTGTTC | DNA corresponding to Pfs28 3' RNA1 |
| Pfs28 RNA1-R | ccgctcgagGAATCATCGAACAAAATATAATTACAAAAG |  |
| Pfs28M RNA1-F | ccgctcgagGAAATGTTCTTTTCCAATTATATTTTGTTC | DNA corresponding to Pfs28 3' RNA1 with mutation |
| Pfs28M RNA1-R | ccgctcgagGAATCATCGAACAAAATATAATTGGAAAAG |  |
| T7-luc-F | TAATACGACTCACTATAGGGctgttggtaaagccaccatggaag | Amplification of DNA template for *in vitro* transcription. T7-luc-F, T7-Pfs25 5' and T7-Pfs28 5' contains T7 promoter sequence and correspond sequences for amplifying DNA templates containing luc ORF and 5' or 3' UTR regions . 50 nt poly A tail was added to reverse primers. |
| T7-Pfs25 5’  T7-Pfs28 5’  PolyA-25 5' | TAATACGACTCACTATAGGGCCGTTTATATAATCATTGTAAAT  TAATACGACTCACTATAGGGATTAAGTTACCTTACTAAGACATG  TTTTTTTTTTTTTTTTTTTTTTTTTTTTTTTTTTTTTTTTTTTT  TTTTTTAAAAGAATAAAAAAATATAAGGTATGAG |  |
| PolyA-25 3' | TTTTTTTTTTTTTTTTTTTTTTTTTTTTTTTTTTTTTTTTTTTT  TTTTTTTAGATATCATTTTATTAGAGTTCATGG |  |
| PolyA-28 5' | TTTTTTTTTTTTTTTTTTTTTTTTTTTTTTTTTTTTTTTTTTTT  TTTTTTTGTATAAAAAACTAAAAAATATAAAA |  |
| PolyA-28 3' | TTTTTTTTTTTTTTTTTTTTTTTTTTTTTTTTTTTTTTTTTTTT  TTTTTTAGAAAACAATATGTTTTTTGCACAAC |  |
| PolyA-pDT 3' | TTTTTTTTTTTTTTTTTTTTTTTTTTTTTTTTTTTTTTTTTTTT  TTTTTTGTCCGATGATGTTGTGGAC |  |
| Hrp2 | GATAGCGATTTTTTTTACTGTCTG | Verification of integration of pLN vector at the Cg6 locus |
| Cg6 | ATGAACAAATACATAAGAGCGC |  |
| Pfs25 5'RACE R1 | CATTTACATTCCAAATGACCACT | RACE to define transcription initiation sites of Pfs25 |
| Pfs25 5'RACE R2 | GCATACAGTATCCACGGTAA |  |
| Pfs25 3'RACE F1 | CCTCTAAAGCTGTTGATGGA | RACE to define transcription termination sites of Pfs25 |
| Pfs25 3'RACE F2 | GAAAGCTCTATATGTACTGCT |  |
| Pfs28 3'RACE F1 | GGAAATACTGTGGACCAGGCT | RACE to define transcription termination sites of Pfs28 |
| Pfs28 3'RACE F2 | GGAGTAACCCTAACACACGTT |  |
| Pfs28 5'RACE R1 | CCTTCAATACACTTACATTCA | RACE to define transcription initiation sites of Pfs28 |
| Pfs28 5'RACE R2 | CGAGCCTTATTCAACGTTATGT |  |
| PfC0495w 5'RACE R1 | GGACATATATGAACAAGAACAC | RACE to define transcription initiation sites of PfC0495w |
| PfC0495w 5'RACE R2 | GGAATATATGGAAGTACGGC |  |
| PfC0495w 3'RACE F1 | CACCATTTTTGATAATGATCATAAAC | RACE to define transcription termination sites of PFC0495w |
| PfC0495w 3'RACE F2 | GGTCTAATTGAAGCTAATCATAAC |  |
| PfL2320w 3'RACE F1 | GTCTATTGGGAATATGACCAATG | RACE to define transcription termination sites of PFL2320w |
| PfL2320w 3'RACE F2 | GGTTACACATAGTAATACTGTTG |  |
| PfL2320w 5'RACE R1 | CATTCTTTGTCTTCTCAAAATCC | RACE to define transcription initiation sites of PfL2320w |
| PfL2320w 5'RACE R2 | GACAGATATGGACTTGTCCTTGG |  |
| hb NRE | AUUAUUUUGUUGUCGAAAAUUGUACAUAAGCC | NRE from *Drosophila* |

­­­
